# SARS-CoV-2 Omicron triggers cross-reactive neutralization and Fc effector functions in previously vaccinated, but not unvaccinated, individuals

**DOI:** 10.1016/j.chom.2022.03.029

**Published:** 2022-06-08

**Authors:** Simone I. Richardson, Vimbai Sharon Madzorera, Holly Spencer, Nelia P. Manamela, Mieke A. van der Mescht, Bronwen E. Lambson, Brent Oosthuysen, Frances Ayres, Zanele Makhado, Thandeka Moyo-Gwete, Nonkululeko Mzindle, Thopisang Motlou, Amy Strydom, Adriano Mendes, Houriiyah Tegally, Zelda de Beer, Talita Roma de Villiers, Annie Bodenstein, Gretha van den Berg, Marietjie Venter, Tulio de Oliviera, Veronica Ueckermann, Theresa M. Rossouw, Michael T. Boswell, Penny L. Moore

**Affiliations:** 1 National Institute for Communicable Diseases of the National Health Laboratory Services, Johannesburg, South Africa; 2 MRC Antibody Immunity Research Unit, School of Pathology, University of the Witwatersrand, Johannesburg, South Africa; 3 Department of Immunology, Faculty of Health Sciences, University of Pretoria, Pretoria, South Africa; 4 Zoonotic Arbo and Respiratory Virus Program, Centre for Viral Zoonoses, Department of Medical Virology, University of Pretoria, Pretoria, South Africa; 5 KwaZulu-Natal Research Innovation and Sequencing Platform, Durban, South Africa; 6 Centre for Epidemic Response and Innovation, School of Data Science and Computational Thinking, Stellenbosch University, Stellenbosch, South Africa; 7 Tshwane District Hospital, Pretoria, South Africa; 8 Division for Infectious Diseases, Department of Internal Medicine, Steve Biko Academic Hospital and University of Pretoria, Pretoria, South Africa; 9 Institute of Infectious Disease and Molecular Medicine, University of Cape Town, Cape Town, South Africa; 10 Centre for the AIDS Programme of Research in South Africa, Durban, South Africa

**Keywords:** SARS-CoV-2, Omicron, neutralization, ADCC, ADCP, variants of concern, vaccines, breakthrough infection, BA.1, BA.2

## Abstract

The SARS-CoV-2 Omicron variant escapes neutralizing antibodies elicited by vaccines or infection. However, whether Omicron triggers cross-reactive humoral responses to other variants of concern (VOCs) remains unknown. We used plasma from 20 unvaccinated and 7 vaccinated individuals infected by Omicron BA.1 to test binding, Fc effector function, and neutralization against VOCs. In unvaccinated individuals, Fc effector function and binding antibodies targeted Omicron and other VOCs at comparable levels. However, Omicron BA.1-triggered neutralization was not extensively cross-reactive for VOCs (14- to 31-fold titer reduction), and we observed 4-fold decreased titers against Omicron BA.2. In contrast, vaccination followed by breakthrough Omicron infection associated with improved cross-neutralization of VOCs with titers exceeding 1:2,100. This has important implications for the vulnerability of unvaccinated Omicron-infected individuals to reinfection by circulating and emerging VOCs. Although Omicron-based immunogens might be adequate boosters, they are unlikely to be superior to existing vaccines for priming in SARS-CoV-2-naive individuals.

## Highlights

• Omicron infection elicits potent neutralizing and Fc effector antibody responses
• Omicron-triggered neutralizing antibodies are poorly cross-reactive for VOCs
• Vaccination prior to infection increases antibody titers and cross-reactivity
• Omicron vaccines are unlikely to be superior to current vaccines in naive individuals

## Graphical abstract

**Figure:**
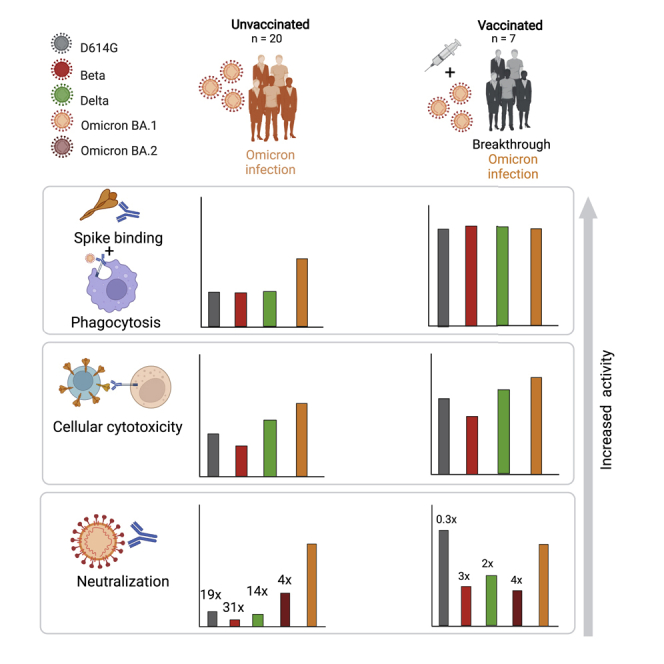

## Teaser

Richardson et al. show that SARS-CoV-2 Omicron infection in unvaccinated individuals triggers potent antibody responses; however, cross-reactivity against variants of concern is poor. In contrast, Omicron BA.1 breakthrough infection in vaccinated individuals elicits high titer cross-reactive antibodies. Omicron-based vaccines are thus unlikely to be superior immunogens in SARS-COV-2-naive individuals.

## Introduction

The emergence of the SARS-CoV-2 Omicron (B.1.1.529) variant of concern (VOC) in November 2021 coincided with the fourth wave of South Africa’s COVID-19 epidemic (Viana et al., 2022). Omicron is defined by multiple mutations across its genome, including more than 30 spike mutations, many of which are associated with immune evasion (Viana et al., 2022). Omicron has further evolved into additional sub-lineages, including BA.1 and BA.2, such that 17 amino acids, 3 deletions, and 1 insertion—many in the N-terminal domain (NTD) and receptor binding domain (RBD)—distinguish the two sub-lineages. These mutations confer neutralization escape in vaccinees and in donors previously infected with other variants (Cele et al., 2021; Garcia-Beltran et al., 2022; Schmidt et al., 2021; Sievers et al., 2022).

In contrast to neutralization, antibody binding to the Omicron variant is preserved, as observed for other VOCs (Carreño et al., 2021; Wibmer et al., 2021). Although Fc receptor binding was substantially reduced in antibodies tested against Omicron (Bartsch et al., 2021), functional Fc effector responses have not yet been reported. The ability of antibodies to bind the Omicron spike suggests that cytotoxic effector functions driven by antibodies might also be retained, as for earlier VOCs (Kaplonek et al., 2022; Richardson et al., 2022). This, along with the finding that T cells triggered by infection or vaccination are cross-reactive for Omicron (Gao et al., 2022; Keeton et al., 2022; Tarke et al., 2022), most likely contributes to maintained vaccine effectiveness against severe disease after Omicron infection (Collie et al., 2022).

Although there is substantial data showing that Omicron evades neutralizing responses, little is known about the humoral response that Omicron infection itself triggers. Defining the capacity of Omicron to trigger cross-reactive binding, neutralizing, and Fc effector antibody responses will inform its potential to protect from reinfection by currently circulating or emerging VOCs. This is of particular relevance for developing countries such as South Africa, where vaccination levels are low and genomic surveillance indicates continued circulation of Delta and other variants. Furthermore, little is known about the activity of antibodies triggered by BA.1 infection against BA.2 (Yamasoba et al., 2022; Yu et al., 2022). Moreover, these data inform the potential immunogenicity of Omicron-based vaccines under development by several companies.

## Results

Plasma from individuals infected in the fourth wave of COVID-19 pandemic in South Africa was used for assessing cross-reactivity against different VOCs for binding, Fc effector function, and neutralization. Plasma was used from 27 hospitalized individuals recruited from Tshwane District Hospital between November 25, 2021, and December 20, 2021, when Omicron BA.1 was responsible for >90% of infections (Viana et al., 2022) (Table S1). Of these, seven plasma samples had matched nasal swabs, and all were Omicron BA.1 infections by sequencing. Twenty individuals were unvaccinated with no history of previous symptomatic COVID-19 infection. Seven individuals had previously been vaccinated with either one dose of Ad26.CoV2.S (n = 2) or two doses of BNT162b2 (n = 5) at least 56 days (56–163 days) prior to infection. Samples were taken a median of four days (1–10 days) after a positive PCR test. The median ages of the vaccinated and unvaccinated individuals were similar (58 and 64 respectively), and infections ranged from mild to severe as determined by World Health Organization (WHO) scoring (Table S1).

**Figure 1:**
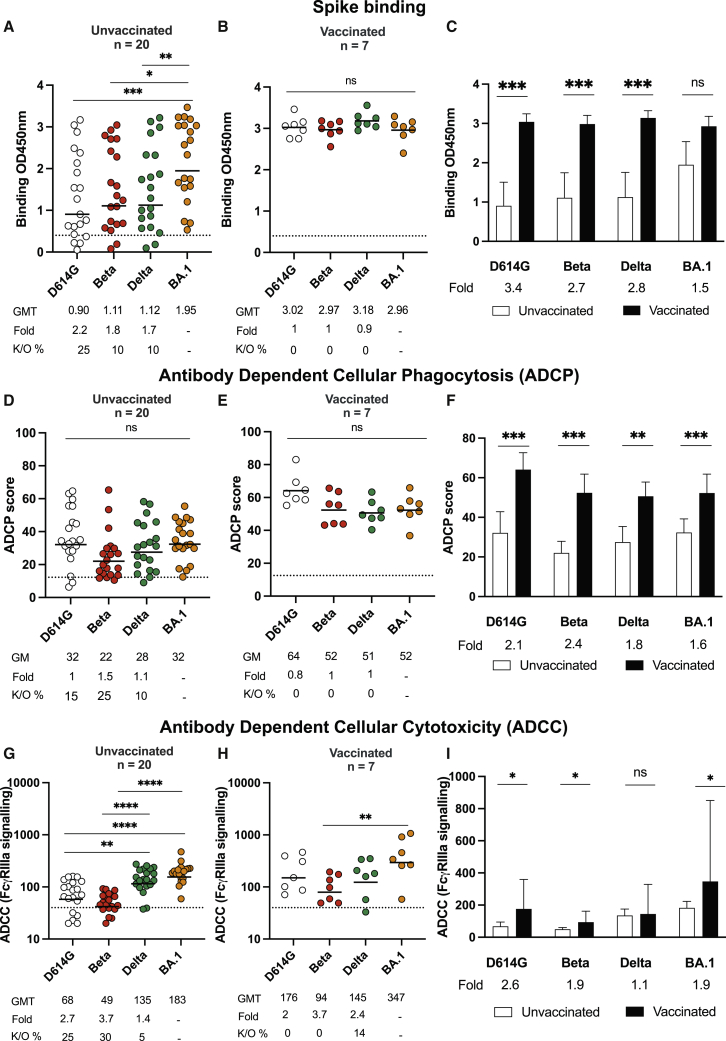
Binding and Fc effector function elicited by Omicron infection is cross-reactive against several variants of concern (VOCs) (A and B) Antibody binding measured by ELISA in (A) unvaccinated individuals (n = 20) or (B) individuals vaccinated with either one dose of Ad26.CoV2.S or two doses of BNT162b2 (n = 7) and infected by Omicron against D614G, Beta, Delta, and Omicron BA.1 spike proteins. (C) Bars show geometric mean (GM) binding titers for vaccinated (black) and unvaccinated (white) individuals against VOCs. (D and E) Antibody-dependent cellular phagocytosis (ADCP) of (D) unvaccinated and (E) vaccinated individuals is represented as the percentage of monocytic cells that take up spike-coated beads (D614G, Beta, Delta, and Omicron BA.1) multiplied by their GM fluorescence intensity (MFI). (F) Bars show GM ADCP scores for vaccinated (black) and unvaccinated (white) individuals against VOCs. (G and H) Antibody-dependent cellular cytotoxicity (ADCC) in (G) unvaccinated and (H) vaccinated individuals is shown as the relative light units (RLU) signaling through FcγRIIIa-expressing cells. (I) Bars show GM activity for vaccinated (black) and unvaccinated (white) individuals against VOCs. All data are representative of two independent experiments. For dot plots, lines indicate GM titer (GMT), which is also represented below the plot with fold decrease and knockout (K/O) of activity for other variants as a percentage relative to Omicron BA.1. Dotted lines indicate the limit of detection of the particular assay. For bar charts, bars indicate the median of function, error bars show standard deviations, and fold decreases relative to vaccinated individuals are indicated below the plot. Statistical significance across variants is shown by the Friedman test with Dunn’s correction, and statistical significance between vaccinated and unvaccinated samples is shown by the Mann Whitney test. ^∗^p < 0.05, ^∗∗^p < 0.01, ^∗∗∗^p < 0.001, ^∗∗∗∗^p < 0.0001, and ns = non-significant.

We first compared levels of binding antibodies, as measured by enzyme-linked immunosorbent assay (ELISA) against the ancestral D614G, Beta, Delta, and Omicron BA.1 spikes. In unvaccinated individuals, titers of binding antibodies against Omicron BA.1 were highest, as expected, and were detectable in all donors. Although we observed statistically significant 2.2-, 1.8-, and 1.7-fold decreases in binding to D614G, Beta, and Delta, respectively, in this group, Omicron BA.1-triggered antibodies were fairly cross-reactive for all variants tested in that they lost activity against other VOCs in 10%–25% of individuals (Figures 1A and 1C). In previously vaccinated individuals who experienced breakthrough infection with Omicron BA.1, binding against Omicron BA.1 was higher than in unvaccinated individuals (geometric mean titer [GMT] of 2.96 versus 1.95) (Figures 1B and 1C). Furthermore, antibodies from these vaccinated individuals exhibited higher levels of cross-reactivity against all variants, and no significant losses were observed (Figure 1B).

Because spike binding antibodies perform Fc effector functions known to contribute to reduced disease severity and vaccine efficacy (Yu et al., 2020; Zohar et al., 2020), we examined antibody-dependent cellular phagocytosis (ADCP) and antibody-dependent cellular cytotoxicity (ADCC) in both groups. For unvaccinated individuals, ADCP against Omicron BA.1 was detected in all 20 individuals with a geometric mean (GM) score of 32 (Figure 1D). Against VOCs, we observed a less than 2-fold reduction in activity across variants: 15%, 25%, and 10% of individuals lost ADCP activity against D614G, and Beta, and Delta, respectively. For vaccinated individuals, non-significant reductions against D614G, Beta, and Delta were observed in relation to Omicron BA.1, and all donors exhibited activity against the panel of VOCs tested here (Figure 1E). Compared with unvaccinated individuals, vaccinated individuals infected with Omicron BA.1 displayed significantly higher levels of ADCP, mirroring the binding antibodies (Figures 1E and 1F).

In contrast to binding and ADCP, ADCC in unvaccinated individuals showed significant losses against D614G (3-fold loss) and Beta (4-fold loss). However, like ADCP and binding antibodies, ADCC activity against Delta was retained (Figure 1G). In this group, Omicron BA.1-triggered ADCC was undetectable against D614G and Beta in 25% and 30% of plasma samples, respectively. After previous vaccination, Omicron BA.1 breakthrough infections resulted in overall preservation of ADCC against VOCs, such that only one individual showed undetectable activity against Delta (Figure 1H). Levels of ADCC in previously vaccinated donors were significantly higher than those in unvaccinated individuals, except that ADCC activity against Delta was similar between both groups (Figure 1I).

**Figure 2:**
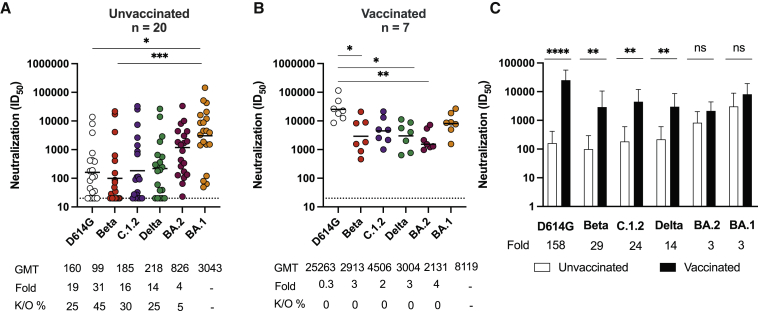
Omicron triggers cross-variant neutralizing antibodies, which are broadened by vaccination (A and B) The neutralization titer (ID_50_) of Omicron-infected plasma against D614G, Beta, C.1.2, Delta, Omicron BA.2, and Omicron BA.1 pseudoviruses is shown for (A) unvaccinated individuals (n = 20) or (B) individuals vaccinated with either one dose of Ad26.CoV.2S or two doses of BNT162b2 (n = 7). Lines indicate GMT, which is also represented below the plot with fold decrease and knockout (K/O) of activity for other variants as a percentage relative to Omicron BA.1. Dotted lines indicate the limit of detection of the assay. Statistical significance across variants is shown by the Friedman test with Dunn’s correction. (C) Bars show GM neutralization titers for vaccinated (black) and unvaccinated (white) individuals against VOCs, error bars show standard deviations, and fold decreases relative to vaccinated individuals are indicated below the plot. Statistical significance between vaccinated and unvaccinated samples is shown by the Mann Whitney test. ^∗^p < 0.05, ^∗∗^p < 0.01, ^∗∗∗^p < 0.001, ^∗∗∗∗^p < 0.0001, and ns = non-significant. All data are representative of two independent experiments.

Lastly, we measured neutralizing antibody responses to Omicron BA.1 and assessed cross-reactivity for VOCs. In addition to testing the variants used above, we also tested C.1.2, a variant that has several neutralization-evasive mutations and circulated at low levels during the third and fourth COVID-19 waves in South Africa (Scheepers et al., 2021), and BA.2, a sub-lineage of Omicron. Against Omicron BA.1, unvaccinated individuals showed potent neutralization with a GMT of 3,043. Neutralization of BA.2 was 4-fold lower than that of BA.1 with a GMT of 826, and only 5% of samples showed knockout of neutralization. However, against VOCs, we saw dramatic 19-, 31-, 16-, and 14-fold titer reductions for D614G, Beta, C.1.2, and Delta, respectively, and knockouts ranging from 27% to 45% of plasma tested (Figure 2A), particularly for Beta. This preferential neutralization of Omicron (the infecting strain) over other VOCs is in sharp contrast to samples from the previous three waves of infection in South Africa (for which the infecting virus was D614G, Beta, or Delta), where neutralizing activity against D614G was substantially higher than that against Omicron BA.1 (Figures S1A and S1B). Similarly, samples from BNT162b2 or Ad26.COV2.S showed higher activity against D614G than against Omicron (Figures S1A and S1B), consistent with previous studies (Cele et al., 2021; Garcia-Beltran et al., 2022).

This loss of neutralization was mitigated by prior vaccination, such that all seven breakthrough infections resulted in a significantly increased GMT of 8,119 against Omicron BA.1, a GMT of 2,131 against Omicron BA.2, and high titers against all VOCs (25,263 for D614G; 2,913 for Beta; 4,506 against C.1.2; and 3,004 against Delta) (Figures 2B and 2C). These greatly enhanced titers resulted in far greater increases between previously vaccinated individuals and unvaccinated individuals (158-, 29-, 24-, 14-, 3-, and 3-fold for D614G, Beta, C.1.2, Delta, Omicron BA.2, and Omicron BA.1 respectively) than those seen for Fc effector functions and binding, which ranged from 1- to 3-fold (Figures 1C, 1F, and 1I). Notably, Omicron infection elicited robust and similar neutralization titers against itself regardless of vaccination status.

## Discussion

Although the neutralization resistance of Omicron is well defined, here we assess how effectively Omicron-elicited antibodies target D614G and other VOCs. We also measured neutralization activity against BA.2, which now accounts for most infections in South Africa and is increasing globally. We show that in previously unvaccinated individuals, Omicron-triggered antibodies bind and perform Fc effector function with only a slight loss against VOCs. However, with the exception of Omicron BA.2, which showed comparatively modest decreases, VOCs significantly compromised neutralization, indicating limited neutralization cross-reactivity of antibodies elicited by Omicron. In contrast, vaccinated individuals who subsequently became infected with Omicron showed greatly improved cross-reactivity with high titers against Omicron BA.1, BA.2, D614G (one amino acid different from the vaccine spike), Beta, Delta, and C.1.2.

We and others have shown that Fc effector function is largely preserved against VOCs in both convalescent and vaccine-elicited plasma (Kaplonek et al., 2022; Richardson et al., 2022). Also, as with neutralization, we have shown that Fc effector function triggered by Beta is more cross-reactive than antibodies elicited by D614G, indicating that the spike sequence of the eliciting immunogen affects the extent of ADCC cross-reactivity (Moyo-Gwete et al., 2021; Richardson et al., 2022). Here, we show that Omicron infection similarly triggers differential ADCC cross-reactivity: significantly decreased activity against D614G and Beta but not against Delta. This observation extends to vaccinated individuals, in whom ADCC was still significantly poorer against Beta. This differential targeting of ADCC-mediating antibodies indicates that they might preferentially bind sites that differ between Omicron and other VOCs. Alternatively, different VOCs might trigger antibodies with varied glycosylations and isotypes, both of which modulate Fc effector function (Jennewein and Alter, 2017).

This differential immune imprinting by VOCs was also confirmed for neutralization in this study in both unvaccinated and vaccinated Omicron-infected individuals. The highest titers in unvaccinated individuals were to the infection-matched Omicron. In contrast, in previously vaccinated individuals with breakthrough Omicron infections, high titers were observed against both D614G and Omicron, sequences that match the vaccine and infecting spike proteins to which these donors were exposed. This is consistent with our studies of immune responses triggered by D614G, Beta, or Delta (Keeton et al., 2021; Moyo-Gwete et al., 2021; Wibmer et al., 2021), which resulted in cross-reactivity patterns distinct from those observed here after Omicron infection. Together, these data indicate that the sequence of the infecting spike affects the quality of neutralization, suggesting imprinting of the immune response (Keeton et al., 2021).

We and others have shown that humoral function is significantly boosted in individuals with breakthrough infections after vaccination (Kitchin et al., 2022; Suryawanshi et al., 2022; Walls et al., 2022). This study confirms that this is also true of Omicron breakthrough infections, as shown by 153-fold higher titers to D614G in vaccinated than in unvaccinated individuals, consistent with other studies (Khan et al., 2022; Suryawanshi et al., 2022; Zhou et al., 2022). We also extend our previous study (in which ADCC was boosted by breakthrough infection) to include ADCP, confirming that this applies to other Fc effector functions (Kitchin et al., 2022).

In the absence of vaccination, Omicron-elicited humoral responses, although potent against the matched Omicron spike, show significantly less activity against VOCs. Thus, although highly immunogenic, Omicron does not elicit cross-neutralizing responses. This is consistent with a decreased ability of plasma from unvaccinated individuals to neutralize Delta compared with Omicron after Omicron infection (Khan et al., 2022), which could leave this unvaccinated group at risk of being reinfected with other variants that continued to circulate and evolve in South Africa at the time of this study, including Beta, Delta, and C.1.2. However, we noted only modestly lower neutralizing titers against Omicron BA.2 than against Omicron BA.1 in this cohort, which is in line with a study showing a 3-fold loss in activity against Omicron BA.2 in Omicron BA.1-infected hamsters (Yamasoba et al., 2022). This indicates that despite a number of differences between the sub-lineages, these changes do not seem to greatly alter the capacity of Omicron BA.1 antibodies to neutralize Omicron BA.2.

Our data also have implications for the design of second-generation vaccines based on Omicron, suggesting that these might not trigger cross-reactive *de novo* responses in SARS-CoV-2-naive individuals. This is supported by immunogenicity studies where Delta-infected mice elicited broadly protective antibodies but where Omicron-infected mice failed to mount responses against other VOCs (Suryawanshi et al., 2022). In addition, immunization of mice with an RBD-based Omicron mRNA vaccine elicited only strain-specific neutralization (Lee et al., 2022). In support of this study and others (Hawman et al., 2022), our data also suggest that Omicron is highly immunogenic in that it elicits comparable neutralization titers irrespectively of vaccination status but through a largely strain-specific response. Given the significantly higher titers we see against Omicron in vaccinated individuals, Omicron boosters could be effective in seropositive individuals, a group that exceeds 70% in South Africa (Madhi et al., 2022). However, in a comparison of mRNA-1273-vaccinated rhesus macaques boosted with either mRNA-Omicron or mRNA-1273, Omicron-boosted animals showed lower titers than those with a homologous mRNA-1273 boost (Gagne et al., 2022). Overall, these data suggest that boosting individuals with or without immunity with vaccines specific to Omicron is unlikely to be superior to existing regimens.

### Limitations of the study

Our study is limited by the fact that we cannot rule out prior asymptomatic infection, which could alter the quality of humoral responses. Sampling for this study was limited to blood and occurred early in infection when antibodies might not have reached peak cross-reactivity. Furthermore, viral sequences confirming Omicron BA.1 infection were available only for a subset of samples. However, Omicron BA.1 overwhelmingly dominated infections during the wave in which these individuals were tested (Viana et al., 2022). Further, the median age of individuals in this study (58 years) is high and could contribute to the compromised cross-reactivity in unvaccinated individuals. Given the high prevalence of global seropositivity through vaccination or previous infection, the ability to measure the response to Omicron infection in naive samples is limited. As such, our study offers valuable insights into the value of Omicron as an immunogen and the risk of reinfection in unvaccinated individuals.

## STAR★Methods

### Key resources table

**Table:** 

REAGENT or RESOURCE	SOURCE	IDENTIFIER
**Antibodies**		

CR3022	GenScript (https://www.genscript.com)	N/A
P2B-2F6	Dr. Nicole Doria-Rose, VRC, USA	N/A
084-7D	this manuscript	N/A
anti-IgG APC (clone QA19A42)	BioLegend	Cat # 366905; RRID: AB_2888847
Palivizumab	Medimmune	Synagis; RRID: AB_2459638

**Bacterial and virus strains**		

SARS-CoV-2 pseudoviruses for ancestral, Beta, Delta, Omicron BA.1, C.1.2, Omicron BA.2	Wibmer et al. (2021), Keeton et al. (2021), Scheepers et al. (2021), this manuscript	N/A

**Biological samples**		

Convalescent hospitalized blood samples	Groote Schuur Hospital	https://www.gsh.co.za
Convalescent hospitalized blood samples	Steve Biko Academic Hospital	https://www.sbah.org.za
Ad26.COV2.S vaccinee blood samples	National institute for Communicable Diseases	https://www.nicd.ac.za
BNT162b2 vaccinee blood samples	National institute for Communicable Diseases	https://www.nicd.ac.za

**Chemicals, peptides, and recombinant proteins**		

SARS-CoV-2 original and Beta variant spike proteins	original: Dr. Jason McKellan; Beta: Moyo-Gwete et al. (2021)	N/A

**Critical commercial assays**		

PEI-MAX 40,000	Polysciences	Cat # 24765–1
QUANTI-Luc luciferase	Invivogen	Cat # rep-qlc2
EZ link Sulfo-NHS-LC-Biotin kit	ThermoFisher	Cat # 21435
FluoSpheres NeutrAvidin-Labeled Microspheres, 1.0 μm	ThermoFisher	Cat # F8776
Luciferase	Promega	Cat # PRE263B-C

**Experimental models: Cell lines**		

Human embryonic kidney (HEK) 293F	Dr. Nicole Doria-Rose, VRC, USA	N/A
HEK 293T/ACE2.MF	Dr. Michael Farzan, Scripps, USA	N/A
Jurkat-Lucia NFAT-CD16 cells	Invivogen	Cat # jktl-nfat-cd16
HEK 293T cells	Dr. George Shaw, UPenn, USA	N/A
THP-1 cells	NIH HIV Reagent program	Cat # ARP-9942

**Recombinant DNA**		

Spike Hexapro plasmid	original: Dr. Jason McKellan; Beta: Moyo-Gwete et al. (2021)	N/A
SARS-CoV-2 ancestral variant spike (D614G) plasmid	Wibmer et al. (2021)	N/A
Beta spike (L18F, D80A, D215G, K417N, E484K, N501Y, D614G, A701V, 242–244 del) plasmid	Wibmer et al. (2021)	N/A
Delta spike (T19R, R158G L452R, T478K, D614G, P681R, D950N, 156–157 del) plasmid	Keeton et al. (2021)	N/A
Omicron BA.1 plasmid (A67V, Δ69-70, T95I, G142D, Δ143-145, Δ211, L212I, 214EPE, G339D, S371L, S373P, S375F, K417N, N440K, G446S, S477N, T478K, E484A, Q493R, G496S, Q498R, N501Y, Y505H, T547K, D614G, H655Y, N679K, P681H, N764K, D796Y, N856K, Q954H, N969K, L981F)	this manuscript	N/A
C.1.2 plasmid (P9L, P25L, C136F, Δ144, R190S, D215G, Δ242-243, Y449H, E484K, N501Y, L585F, D614G, H655Y, N679K, T716I, T859N)	Scheepers et al. (2021)	N/A
Omicron BA.2 plasmid (T19I, L24S, 25–27del, G142D, V213G, G339D, S371F, S373P, S375F, T376A, D405N, R408S,K417N,N440K, S477N, T478K, E484A, Q493R, Q498R, N501Y, Y505H, D614G, H655Y, N679K, P681H, N764K, D796Y, Q954H, N969K)	this manuscript	N/A
Firefly luciferase encoding lentivirus backbone plasmid	Dr. Michael Farzan, Scripps	N/A

**Software and algorithms**		

Genome Detective 1.132	Genome Detective	https://www.genomedetective.com
Geneious software	Biomatters	https://www.geneious.com
NextStrain	Hadfield et al. (2018)	https://github.com/nextstrain/ncov
FACSDiva 9	BD Biosciences	https://www.bdbiosciences.com
FlowJo 10	FlowJo	https://www.flowjo.com
GraphPad Prism 9	GraphPad	https://www.graphpad.com
BioRender	BioRender	https://www.biorender.com

### Resource availability

#### Lead contact

Further information and reasonable requests for resources and reagents should be directed to and will be fulfilled by the lead contact, Penny Moore (pennym@nicd.ac.za).

#### Materials availability

Materials will be made by request to Penny Moore (pennym@nicd.ac.za).

### Experimental model and subject details

#### Human subjects

Samples infected in the fourth COVID-19 wave of infection in South Africa were collected from participants enrolled to the Pretoria COVID-19 study cohort. Participants were admitted to Tshwane District Hospital (Pretoria, South Africa) with mild to severe (WHO severity scale 3–6) PCR confirmed SARS-CoV-2 infection between 25 November 2021–20 December 2021 (Table S1). Ethics approval was received from the University of Pretoria, Human Research Ethics Committee (Medical) (247/2020). Second wave plasma samples were obtained from hospitalized COVID-19 patients with moderate disease (WHO scale 4–5) admitted to Groote Schuur Hospital cohort, Cape Town from December 2020 to January 2021. This study received ethics approval from the Human Research Ethics Committee of the Faculty of Health Sciences, University of Cape Town (R021/2020). Wave 3 samples were collected from hospitalized COVID-19 patients with moderate disease (WHO scale 4–5) from Steve Biko Academic Hospital and Groote Schuur Hospital in July 2021. All patients had PCR-confirmed SARS-CoV-2 infection before blood collection, which was done a median of 4 days post positive PCR test. Healthy donors who were confirmed to be SARS-CoV-2 infection naive and were vaccinated with either one dose of Janssen/Johnson and Johnson (Ad26.COV2.S) vaccine during the Sisonke Trial or two doses of BNT162b2 were obtained 2 months after full vaccination. Ethics approval for the use of these samples were obtained from the Human Research Ethics Committee of the Faculty of Health Sciences, University of the Witwatersrand (M210465). Written informed consent was obtained from all participants.

#### Cell lines

Human embryo kidney (HEK) 293T cells were cultured at 37°C and 5% CO_2_ in DMEM containing 10% heat-inactivated fetal bovine serum (Gibco BRL Life Technologies) and supplemented with 50 μg/mL gentamicin (Sigma). Cells were disrupted at confluence with 0.25% trypsin in 1 mM EDTA (Sigma) every 48–72 h. HEK 293T/ACE2.MF cells were maintained in the same way as HEK 293T cells but were supplemented with 3 μg/mL puromycin for selection of stably transduced cells. HEK 293F suspension cells were cultured in 293 Freestyle media (Gibco BRL Life Technologies) and cultured in a shaking incubator at 37°C, 5% CO_2_, and 70% humidity at 125 rpm maintained between 0.2 and 0.5 million cells/mL. Jurkat-Lucia NFAT-CD16 cells were maintained in IMDM media with 10% heat-inactivated fetal bovine serum (Gibco, Gaithersburg, MD), 1% penicillin streptomycin (Gibco, Gaithersburg, MD) and 10 μg/mL of blasticidin and 100 μg/mL of zeocin was added to the growth medium every other passage. THP-1 cells were used for the ADCP assay and obtained from the AIDS Reagent Program, Division of AIDS, NIAID, NIH contributed by Dr. Li Wu and Vineet N. KewalRamani. Cells were cultured at 37°C and 5% CO_2_ in RPMI containing 10% heat-inactivated fetal bovine serum (Gibco, Gaithersburg, MD) with 1% Penicillin Streptomycin (Gibco, Gaithersburg, MD) and 2-mercaptoethanol to a final concentration of 0.05 mM and not allowed to exceed 4 × 10^5^ cells/mL to prevent differentiation.

### Method details

#### SARS-CoV-2 spike genome sequencing

Sequencing of the spike was performed as previously described (Tegally et al., 2021) using swabs obtained from Tshwane District Hospital patients, of which six were available and confirmed to be Omicron BA.1 (Table S1). RNA sequencing was performed as previously published. Briefly, extracted RNA was used to synthesize cDNA using the Superscript IV First Strand synthesis system (Life Technologies, Carlsbad, CA) and random hexamer primers. SARS-CoV-2 whole genome amplification was performed by multiplex PCR using primers designed on Primal Scheme (http://primal.zibraproject.org/) to generate 400 bp amplicons with a 70 bp overlap covering the SARS-CoV-2 genome. Phylogenetic clade classification of the genomes in this study consisted of analyzing them against a global reference dataset using a custom pipeline based on a local version of NextStrain (https://github.com/nextstrain/ncov) (Hadfield et al., 2018).

#### SARS-CoV-2 antigens

For ELISA and ADCP assays, SARS-CoV-2 original and Beta variant full spike (L18F, D80A, D215G, K417N, E484K, N501Y, D614G, A701V, 242–244 del), Delta (T19R, 156–157del, R158G, L452R, T478K, D614G, P681R and D950N) and Omicron BA.1 (A67V, Δ69-70, T95I, G142D, Δ143-145, Δ211, L212I, 214EPE, G339D, S371L, S373P, S375F, K417N, N440K, G446S, S477N, T478K, E484A, Q493R, G496S, Q498R, N501Y, Y505H, T547K, D614G, H655Y, N679K, P681H, N764K, D796Y, N856K, Q954H, N969K, L981F) proteins were expressed in HEK 293F suspension cells by transfecting the cells with the respective expression plasmid. After incubating for 6 days at 37°C, 70% humidity, and 10% CO_2_, proteins were first purified using a nickel resin followed by size-exclusion chromatography. Relevant fractions were collected and frozen at −80°C until use.

#### SARS-CoV-2 spike ELISA

Two μg/mL of spike protein (D614G, Beta, Delta, or Omicron) was used to coat 96-well, high-binding plates and incubated overnight at 4°C. The plates were incubated in a blocking buffer consisting of 5% skimmed milk powder, 0.05% Tween 20, 1× PBS. Plasma samples were diluted to 1:100 starting dilution in a blocking buffer and added to the plates. IgG secondary antibody was diluted to 1:3000 in blocking buffer and added to the plates followed by TMB substrate (Thermofisher Scientific). Upon stopping the reaction with 1 M H_2_SO_4_, absorbance was measured at a 450 nm wavelength. In all instances, mAbs CR3022 and BD23 were used as positive controls and Palivizumab was used as a negative control.

#### Spike plasmid and lentiviral pseudovirus production

The SARS-CoV-2 Wuhan-1 spike, cloned into pCDNA3.1 was mutated using the QuikChange Lightning Site-Directed Mutagenesis kit (Agilent Technologies) and NEBuilder HiFi DNA Assembly Master Mix (NEB) to include D614G (original) or lineage defining mutations for Beta (L18F, D80A, D215G, 242–244del, K417N, E484K, N501Y, D614G and A701V), Delta (T19R, 156–157del, R158G, L452R, T478K, D614G, P681R and D950N), C.1.2 (P9L, P25L, C136F, Δ144, R190S, D215G, Δ242-243, Y449H, E484K, N501Y, L585F, D614G, H655Y, N679K, T716I, T859N), Omicron BA.1 (A67V, Δ69-70, T95I, G142D, Δ143-145, Δ211, L212I, 214EPE, G339D, S371L, S373P, S375F, K417N, N440K, G446S, S477N, T478K, E484A, Q493R, G496S, Q498R, N501Y, Y505H, T547K, D614G, H655Y, N679K, P681H, N764K, D796Y, N856K, Q954H, N969K, L981F) or Omicron BA.2 (T19I, L24S, 25–27del, G142D, V213G, G339D, S371F, S373P, S375F, T376A, D405N, R408S,K417N,N440K, S477N, T478K, E484A, Q493R, Q498R, N501Y, Y505H, D614G, H655Y, N679K, P681H, N764K, D796Y, Q954H, N969K).

Pseudotyped lentiviruses were prepared by co-transfecting HEK 293T cell line with the SARS-CoV-2 ancestral variant spike (D614G), Beta, Delta, C.1.2, Omicron BA.1, or Omicron BA.2 spike plasmids in conjunction with a firefly luciferase encoding lentivirus backbone (HIV-1 pNL4.luc) plasmid as previously described (Wibmer et al., 2021). Culture supernatants were clarified of cells by a 0.45-μM filter and stored at −70°C. Other pcDNA plasmids were used for the ADCC assay.

#### Pseudovirus neutralization assay

For the neutralization assay, plasma samples were heat-inactivated and clarified by centrifugation. Heat-inactivated plasma samples from vaccine recipients were incubated with the SARS-CoV-2 pseudotyped virus for 1 h at 37°C and 5% CO_2_. Subsequently, 1 × 10^4^ HEK 293T cells engineered to over-express ACE-2 (293T/ACE2.MF) (kindly provided by M. Farzan, Scripps Research) were added and incubated at 37°C and 5% CO_2_ for 72 h, upon which the luminescence of the luciferase gene was measured. Titers were calculated as the reciprocal plasma dilution (ID_50_) causing 50% reduction of relative light units. CB6 and CA1 was used as positive controls for D614G, Beta and Delta. 084-7D, a mAb targeting K417N was used as a positive control for Omicron and Beta.

#### ADCP assay

Avitagged SARS-CoV-2 spikes were biotinylated using the BirA biotin-protein ligase standard reaction kit (Avidity) and coated onto fluorescent neutravidin beads as previously described (Ackerman et al., 2011). In brief, beads were incubated for two hours with monoclonal antibodies at a starting concentration of 20 μg/mL or plasma at a single 1 in 100 dilution. Opsonized beads were incubated with the monocytic THP-1 cell line overnight, fixed and interrogated on the FACSAria II. Phagocytosis score was calculated as the percentage of THP-1 cells that engulfed fluorescent beads multiplied by the GM fluorescence intensity of the population less the no antibody control. For this and all subsequent Fc effector assays, pooled plasma from 5 PCR-confirmed SARS-CoV-2 infected individuals and CR3022 were used as positive controls and plasma from 5 pre-pandemic healthy controls and Palivizumab were used as negative controls. 084-7D was used as a positive control for Omicron BA.1 and Beta. In addition samples both waves were run head to head in the same experiment. ADCP scores for different spikes were normalized to each other and between runs using CR3022.

#### ADCC assay

The ability of plasma antibodies to cross-link and signal through FcγRIIIa (CD16) and spike expressing cells or SARS-CoV-2 protein was measured as a proxy for ADCC. For spike assays, HEK 293T cells were transfected with 5 μg of SARS-CoV-2 spike plasmids using PEI-MAX 40,000 (Polysciences) and incubated for 2 days at 37°C. Expression of spike was confirmed by differential binding of CR3022 and P2B-2F6 and their detection by anti-IgG APC staining measured by flow cytometry. Subsequently, 1 × 10^5^ spike transfected cells per well were incubated with heat inactivated plasma (1:100 final dilution) or monoclonal antibodies (final concentration of 100 μg/mL) in RPMI 1640 media supplemented with 10% FBS 1% Pen/Strep (Gibco, Gaithersburg, MD) for 1 h at 37°C. Jurkat-Lucia NFAT-CD16 cells (Invivogen) (2 × 10^5^ cells/well and 1 × 10^5^ cells/well for spike and other protein respectively) were added and incubated for 24 h at 37°C and 5% CO_2_. Twenty μL of supernatant was then transferred to a white 96-well plate with 50 μL of reconstituted QUANTI-Luc secreted luciferase and read immediately on a Victor 3 luminometer with 1 s integration time. Relative light units (RLU) of a no antibody control was subtracted as background. Palivizumab was used as a negative control, while CR3022 was used as a positive control, and P2B-2F6 to differentiate the Beta from the D614G variant. 084-7D was used as a positive control for Omicron BA.1 and Beta. To induce the transgene 1× cell stimulation cocktail (Thermofisher Scientific, Oslo, Norway) and 2 μg/mL ionomycin in R10 was added as a positive control to confirm sufficient expression of the Fc receptor. RLUs for spikes were normalized to each other and between runs using CR3022. All samples were run head to head in the same experiment as were all variants tested.

### Quantification and statistical analysis

Analyses were performed in Prism (v9; GraphPad Software, San Diego, CA, USA). Non-parametric tests were used for all comparisons. The Mann-Whitney and Wilcoxon tests were used for unmatched and paired samples, respectively. The Friedman test with Dunn’s correction for multiple comparisons was used for matched comparisons across variants. All correlations reported are non-parametric Spearman’s correlations. p values less than 0.05 were considered to be statistically significant.

## Data Availability

• All data reported in this paper will be shared by the lead contact upon request. • This paper does not report any original code. • Any additional information required for reanalyzing the data reported in this paper is available from the lead contact upon request.

## Supplemental information

Document S1. Figure S1 and Table S1

Document S2. Article plus supplemental information
