# Supplementary material for: SARS-CoV-2 Omicron triggers cross-reactive neutralization and Fc effector functions in previously vaccinated, but not unvaccinated, individuals
Source: Cell Host Microbe. 2022 Jun 8;30(6):880–886.e4. doi: 10.1016/j.chom.2022.03.029 (PMC8947963; doi:10.1016/j.chom.2022.03.029)
Supplement: Document S1. Figure S1 and Table S1 [file mmc1.pdf]

Cell Host & Microbe, Volume *30*

Supplemental information

SARS-CoV-2 Omicron triggers cross-reactive neutralization and Fc effector functions in previously vaccinated, but not unvaccinated, individuals

Simone I. Richardson, Vimbai Sharon Madzorera, Holly Spencer, Nelia P. Manamela, Mieke A. van der Mescht, Bronwen E. Lambson, Brent Oosthuysen, Frances Ayres, Zanele Makhado, Thandeka Moyo-Gwete, Nonkululeko Mzindle, Thopisang Motlou, Amy Strydom, Adriano Mendes, Houriiyah Tegally, Zelda de Beer, Talita Roma de Villiers, Annie Bodenstein, Gretha van den Berg, Marietjie Venter, Tulio de Oliviera, Veronica Ueckermann, Theresa M. Rossouw, Michael T. Boswell, and Penny L. Moore

Table S1: Demographic and clinical description of the cohort, Related to STAR Methods

Study number	Age	Gender	Positive PCR test	Days sampled post infection	Vaccination status	Days since vaccination	Sequence	Severity	WHO severity scale
COV132	36	Male	2021/11/28	9	Unvaccinated	-	Not sequenced	Severe	6
COV140	29	Female	2021/12/09	1	Unvaccinated	-	Not sequenced	Mild	2
COV145	17	Female	2021/12/03	3	Unvaccinated	-	Omicron	Mild	2
COV149	63	Female	2021/12/05	3	Unvaccinated	-	Not sequenced	Mild	2
COV157	66	Male	2021/12/04	4	Unvaccinated	-	Omicron	Moderate	5
COV158	69	Female	2021/12/14	6	Unvaccinated	-	Not sequenced	Moderate	5
COV159	24	Male	2021/11/25	6	Unvaccinated	-	Not sequenced	Mild	2
COV162	58	Male	2021/12/09	5	Unvaccinated	-	Not sequenced	Moderate	5
COV165	62	Female	2021/12/10	4	Unvaccinated	-	Not sequenced	Moderate	5
COV166	59	Male	2021/12/10	4	Unvaccinated	-	Not sequenced	Severe	6
COV167	62	Female	2021/11/29	8	Unvaccinated	-	Not sequenced	Severe	5
COV176	75	Male	2021/12/20	2	Unvaccinated	-	Not sequenced	Moderate	6
COV178	61	Female	2021/12/19	2	Unvaccinated	-	Not sequenced	Moderate	5
COV183	27	Male	2021/12/12	2	Unvaccinated	-	Not sequenced	Mild	2
COV184	59	Male	2021/12/14	1	Unvaccinated	-	Not sequenced	Moderate	5
COV185	47	Female	2021/12/13	1	Unvaccinated	-	Not sequenced	Moderate	5
COV186	58	Male	2021/12/03	10	Unvaccinated	-	Omicron	Moderate	5
COV187	35	Female	2021/12/11	3	Unvaccinated	-	Not sequenced	Mild	2
COV189	49	Male	2021/12/09	5	Unvaccinated	-	Not sequenced	Moderate	5
COV190	34	Female	2021/12/10	4	Unvaccinated	-	Omicron	Mild	2
COV151	48	Male	2021/12/07	1	One dose Ad26.CoV2.S	56	Omicron	Severe	5
COV182	39	Male	2021/12/10	3	One dose Ad26.CoV2.S	Unknown	Not sequenced	Mild	2
COV131	59	Female	2021/12/10	4	Two doses BNT162b2	109	Not sequenced	Severe	6
COV135	85	Male	2021/11/26	5	Two doses BNT162b2	72	Omicron	Mild	2
COV138	68	Male	2021/11/25	5	Two doses BNT162b2	Unknown	Not sequenced	Moderate	3
COV161	64	Female	2021/12/05	5	Two doses BNT162b2	112	Not sequenced	Mild	2
COV181	71	Male	2021/12/16	4	Two doses BNT162b2	163	Not sequenced	Severe	6

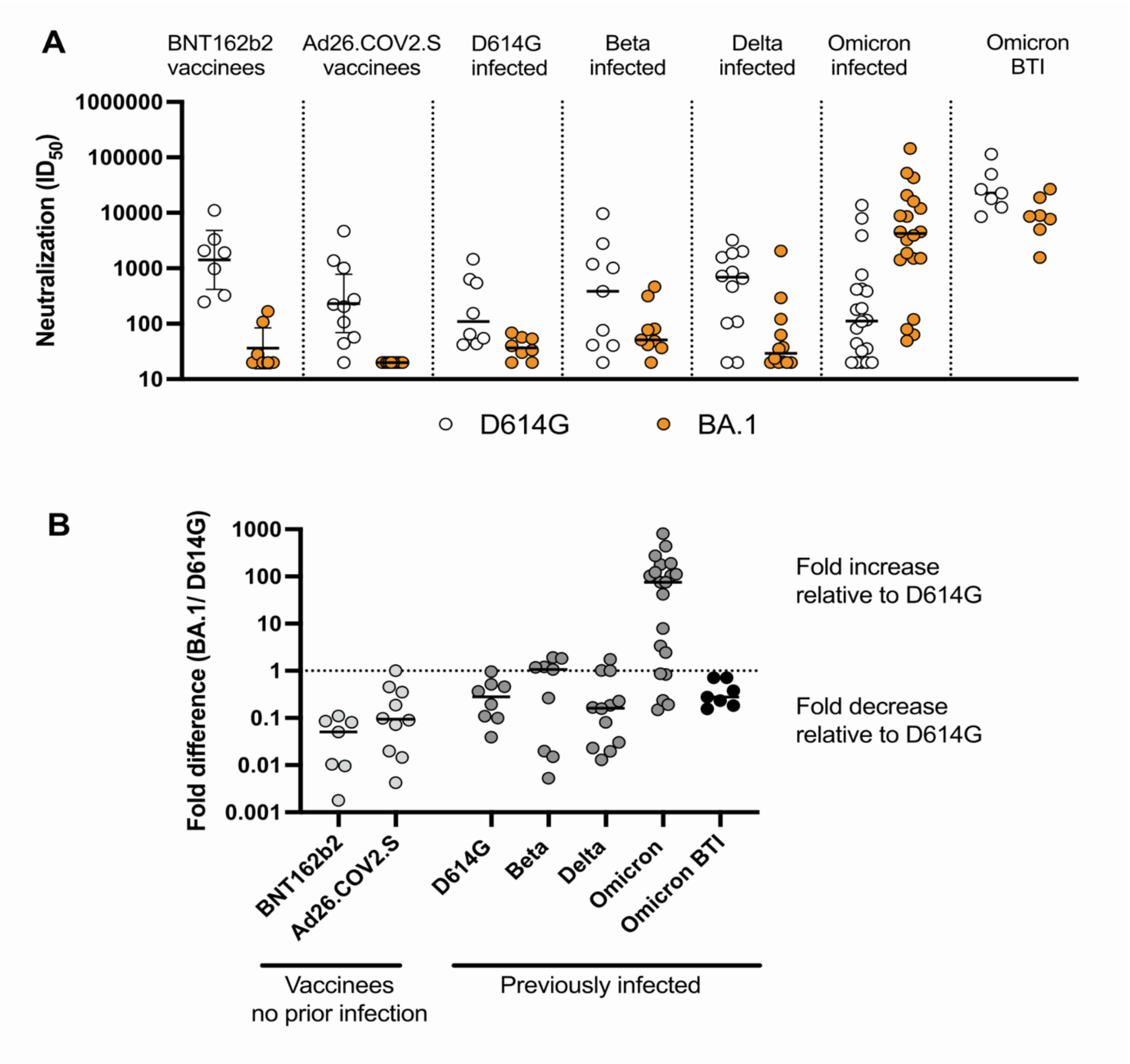

Figure S1: Omicron BA.1 neutralization titers are substantially boosted in response to Omicron infection compared to infection with other VOCs or vaccination, Related to Figure 2

(A) Neutralization ID_{50} titers from vaccinees two months post two-doses of BNT162b2 or one-dose of Ad26.COV2.S, individuals acutely infected with D614G, Beta, Delta or Omicron or individuals who were vaccinated with either BNT162b2 or Ad26.COV2.S and then infected with Omicron (BTI = breakthrough infection) against D614G (white) and Omicron BA.1 (orange) SARS-CoV-2 variants. Lines indicate geometric mean titer (GMT). (B) Fold difference of neutralization titer against Omicron BA.1 relative to D614G for each individual is shown with median fold difference indicated by the bold line. The dotted line indicates no fold difference.
