# Supplementary material for: SARS-CoV-2 Omicron triggers cross-reactive neutralization and Fc effector functions in previously vaccinated, but not unvaccinated, individuals
Source: Cell Host Microbe. 2022 Jun 8;30(6):880–886.e4. doi: 10.1016/j.chom.2022.03.029 (PMC8947963; doi:10.1016/j.chom.2022.03.029)
Supplement: Document S2. Article plus supplemental information [file mmc2.pdf]

Brief Report

Cell Host & Microbe

SARS-CoV-2 Omicron triggers cross-reactive neutralization and Fc effector functions in previously vaccinated, but not unvaccinated, individuals

Graphical abstract

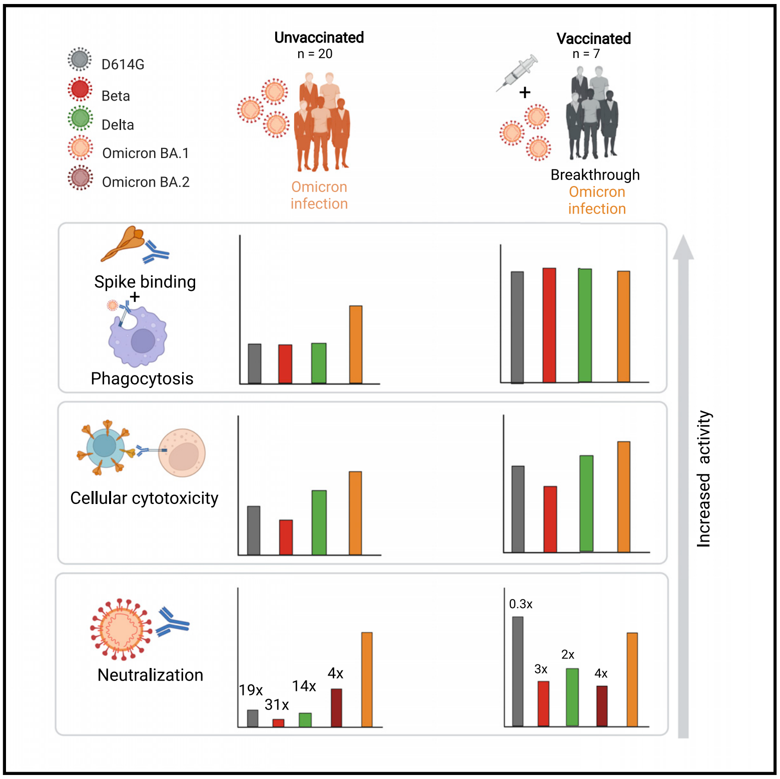

Authors

Simone I. Richardson, Vimbai Sharon Madzorera, Holly Spencer, ..., Theresa M. Rossouw, Michael T. Boswell, Penny L. Moore

Correspondence

pennym@nicd.ac.za

In brief

Richardson et al. show that SARS-CoV-2 Omicron infection in unvaccinated individuals triggers potent antibody responses; however, cross-reactivity against variants of concern is poor. In contrast, Omicron BA.1 breakthrough infection in vaccinated individuals elicits high titer cross-reactive antibodies. Omicron-based vaccines are thus unlikely to be superior immunogens in SARS-COV-2-naive individuals.

Highlights

- Omicron infection elicits potent neutralizing and Fc effector antibody responses
- Omicron-triggered neutralizing antibodies are poorly cross-reactive for VOCs
- Vaccination prior to infection increases antibody titers and cross-reactivity
- Omicron vaccines are unlikely to be superior to current vaccines in naive individuals

Richardson et al., 2022, Cell Host & Microbe *30*, 880–886
June 8, 2022 © 2022 The Authors. Published by Elsevier Inc.
https://doi.org/10.1016/j.chom.2022.03.029

CellPress

CellPress
OPEN ACCESS

Cell Host & Microbe

Brief Report

SARS-CoV-2 Omicron triggers cross-reactive neutralization and Fc effector functions in previously vaccinated, but not unvaccinated, individuals

Simone I. Richardson,[1,2] Vimbai Sharon Madzorera,[1,2] Holly Spencer,[1,2] Nelia P. Manamela,[1,2] Mieke A. van der Mescht,[3] Bronwen E. Lambson,[1,2] Brent Oosthuysen,[1,2] Frances Ayres,[1,2] Zanele Makhado,[1,2] Thandeka Moyo-Gwete,[1,2] Nonkululeko Mzindle,[1,2] Thopisang Motlou,[1,2] Amy Strydom,[4] Adriano Mendes,[4] Houriiyah Tegally,[5,6] Zelda de Beer,[7] Talita Roma de Villiers,[7] Annie Bodenstein,[7] Gretha van den Berg,[7] Marietjie Venter,[4] Tulio de Oliviera,[5,6,10] Veronica Ueckermann,[8] Theresa M. Rossouw,[3] Michael T. Boswell,[8] and Penny L. Moore[1,2,9,10,11,*]

[1]National Institute for Communicable Diseases of the National Health Laboratory Services, Johannesburg, South Africa
[2]MRC Antibody Immunity Research Unit, School of Pathology, University of the Witwatersrand, Johannesburg, South Africa
[3]Department of Immunology, Faculty of Health Sciences, University of Pretoria, Pretoria, South Africa
[4]Zoonotic Arbo and Respiratory Virus Program, Centre for Viral Zoonoses, Department of Medical Virology, University of Pretoria, Pretoria, South Africa
[5]KwaZulu-Natal Research Innovation and Sequencing Platform, Durban, South Africa
[6]Centre for Epidemic Response and Innovation, School of Data Science and Computational Thinking, Stellenbosch University, Stellenbosch, South Africa
[7]Tshwane District Hospital, Pretoria, South Africa
[8]Division for Infectious Diseases, Department of Internal Medicine, Steve Biko Academic Hospital and University of Pretoria, Pretoria, South Africa
[9]Institute of Infectious Disease and Molecular Medicine, University of Cape Town, Cape Town, South Africa
[10]Centre for the AIDS Programme of Research in South Africa, Durban, South Africa
[11]Lead contact
*Correspondence: pennym@nicd.ac.za
https://doi.org/10.1016/j.chom.2022.03.029

SUMMARY

The SARS-CoV-2 Omicron variant escapes neutralizing antibodies elicited by vaccines or infection. However, whether Omicron triggers cross-reactive humoral responses to other variants of concern (VOCs) remains unknown. We used plasma from 20 unvaccinated and 7 vaccinated individuals infected by Omicron BA.1 to test binding, Fc effector function, and neutralization against VOCs. In unvaccinated individuals, Fc effector function and binding antibodies targeted Omicron and other VOCs at comparable levels. However, Omicron BA.1-triggered neutralization was not extensively cross-reactive for VOCs (14- to 31-fold titer reduction), and we observed 4-fold decreased titers against Omicron BA.2. In contrast, vaccination followed by breakthrough Omicron infection associated with improved cross-neutralization of VOCs with titers exceeding 1:2,100. This has important implications for the vulnerability of unvaccinated Omicron-infected individuals to reinfection by circulating and emerging VOCs. Although Omicron-based immunogens might be adequate boosters, they are unlikely to be superior to existing vaccines for priming in SARS-CoV-2-naive individuals.

INTRODUCTION

The emergence of the SARS-CoV-2 Omicron (B.1.1.529) variant of concern (VOC) in November 2021 coincided with the fourth wave of South Africa's COVID-19 epidemic (Viana et al., 2022). Omicron is defined by multiple mutations across its genome, including more than 30 spike mutations, many of which are associated with immune evasion (Viana et al., 2022). Omicron has further evolved into additional sub-lineages, including BA.1 and BA.2, such that 17 amino acids, 3 deletions, and 1 insertion—many in the N-terminal domain (NTD) and receptor binding domain (RBD)—distinguish the two sub-lineages. These mutations confer neutralization escape in vaccinees and in donors previously infected with other variants (Cele et al., 2021; Garcia-Beltran et al., 2022; Schmidt et al., 2021; Sievers et al., 2022).

In contrast to neutralization, antibody binding to the Omicron variant is preserved, as observed for other VOCs (Carreño et al., 2021; Wibmer et al., 2021). Although Fc receptor binding was substantially reduced in antibodies tested against Omicron (Bartsch et al., 2021), functional Fc effector responses have not yet been reported. The ability of antibodies to bind the Omicron spike suggests that cytotoxic effector functions driven by antibodies might also be retained, as for earlier VOCs (Kaplonek et al., 2022; Richardson et al., 2022). This, along with the finding that T cells triggered by infection or vaccination are cross-reactive for Omicron (Gao et al., 2022; Keeton et al., 2022; Tarke

 © 2022 The Authors. Published by Elsevier Inc.
This is an open access article under the CC BY license (http://creativecommons.org/licenses/by/4.0/).

CellPress
OPEN ACCESS

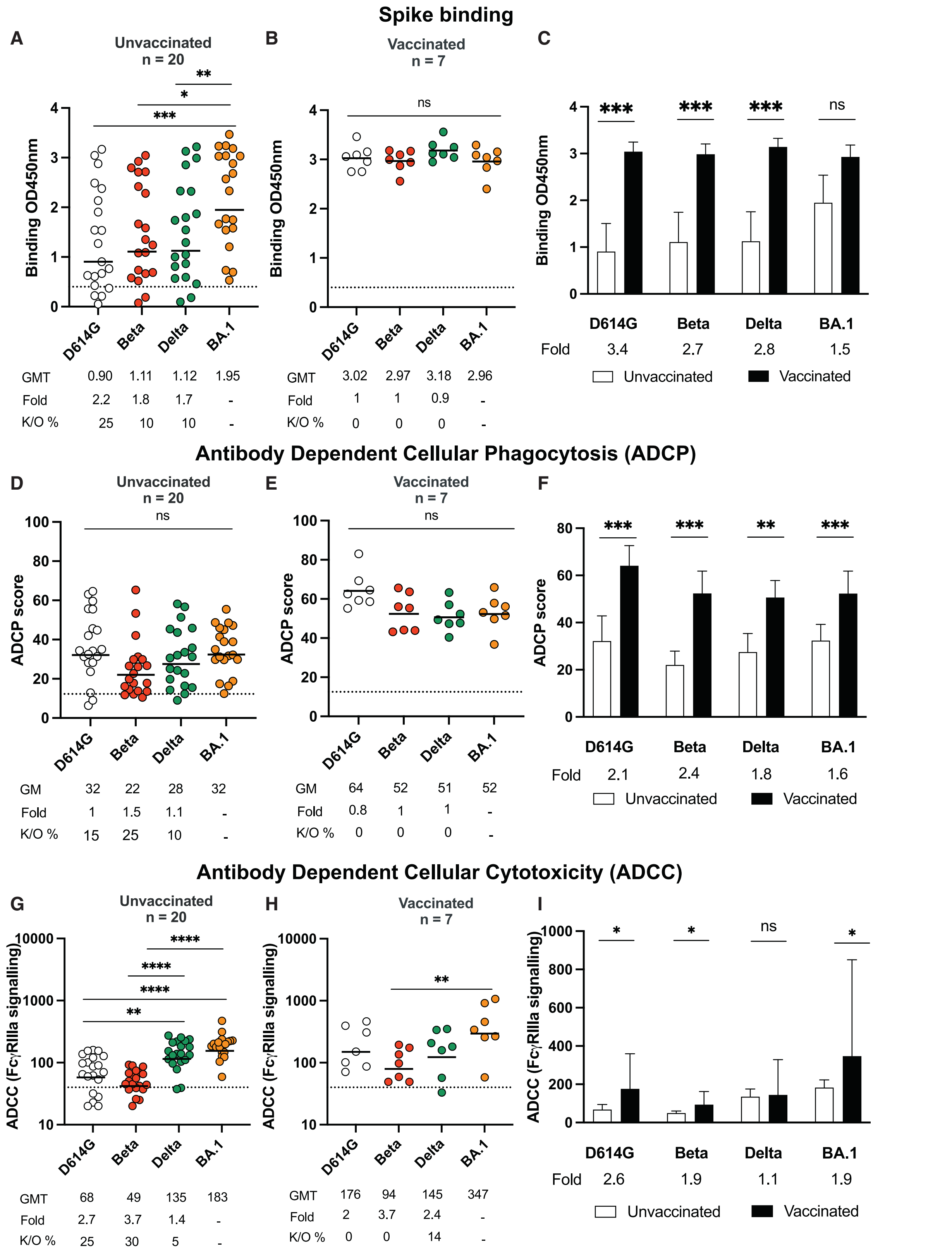

Figure 1. Binding and Fc effector function elicited by Omicron infection is cross-reactive against several variants of concern (VOCs)

(A and B) Antibody binding measured by ELISA in (A) unvaccinated individuals (n = 20) or (B) individuals vaccinated with either one dose of Ad26.CoV2.S or two doses of BNT162b2 (n = 7) and infected by Omicron against D614G, Beta, Delta, and Omicron BA.1 spike proteins.

(legend continued on next page)

et al., 2022), most likely contributes to maintained vaccine effectiveness against severe disease after Omicron infection (Collie et al., 2022).

Although there is substantial data showing that Omicron evades neutralizing responses, little is known about the humoral response that Omicron infection itself triggers. Defining the capacity of Omicron to trigger cross-reactive binding, neutralizing, and Fc effector antibody responses will inform its potential to protect from reinfection by currently circulating or emerging VOCs. This is of particular relevance for developing countries such as South Africa, where vaccination levels are low and genomic surveillance indicates continued circulation of Delta and other variants. Furthermore, little is known about the activity of antibodies triggered by BA.1 infection against BA.2 (Yamasoba et al., 2022; Yu et al., 2022). Moreover, these data inform the potential immunogenicity of Omicron-based vaccines under development by several companies.

RESULTS

Plasma from individuals infected in the fourth wave of COVID-19 pandemic in South Africa was used for assessing cross-reactivity against different VOCs for binding, Fc effector function, and neutralization. Plasma was used from 27 hospitalized individuals recruited from Tshwane District Hospital between November 25, 2021, and December 20, 2021, when Omicron BA.1 was responsible for >90% of infections (Viana et al., 2022) (Table S1). Of these, seven plasma samples had matched nasal swabs, and all were Omicron BA.1 infections by sequencing. Twenty individuals were unvaccinated with no history of previous symptomatic COVID-19 infection. Seven individuals had previously been vaccinated with either one dose of Ad26.CoV2.S (n = 2) or two doses of BNT162b2 (n = 5) at least 56 days (56–163 days) prior to infection. Samples were taken a median of four days (1–10 days) after a positive PCR test. The median ages of the vaccinated and unvaccinated individuals were similar (58 and 64 respectively), and infections ranged from mild to severe as determined by World Health Organization (WHO) scoring (Table S1).

We first compared levels of binding antibodies, as measured by enzyme-linked immunosorbent assay (ELISA) against the ancestral D614G, Beta, Delta, and Omicron BA.1 spikes. In unvaccinated individuals, titers of binding antibodies against Omicron BA.1 were highest, as expected, and were detectable in all donors. Although we observed statistically significant 2.2-, 1.8-, and 1.7-fold decreases in binding to D614G, Beta, and Delta, respectively, in this group, Omicron BA.1-triggered antibodies were fairly cross-reactive for all variants tested in that they lost activity against other VOCs in 10%–25% of individuals (Figures 1A and 1C). In previously vaccinated individuals who experienced breakthrough infection with Omicron BA.1, binding against Omicron BA.1 was higher than in unvaccinated individuals (geometric mean titer [GMT] of 2.96 versus 1.95) (Figures 1B and 1C). Furthermore, antibodies from these vaccinated individuals exhibited higher levels of cross-reactivity against all variants, and no significant losses were observed (Figure 1B).

Because spike binding antibodies perform Fc effector functions known to contribute to reduced disease severity and vaccine efficacy (Yu et al., 2020; Zohar et al., 2020), we examined antibody-dependent cellular phagocytosis (ADCP) and antibody-dependent cellular cytotoxicity (ADCC) in both groups. For unvaccinated individuals, ADCP against Omicron BA.1 was detected in all 20 individuals with a geometric mean (GM) score of 32 (Figure 1D). Against VOCs, we observed a less than 2-fold reduction in activity across variants: 15%, 25%, and 10% of individuals lost ADCP activity against D614G, and Beta, and Delta, respectively. For vaccinated individuals, non-significant reductions against D614G, Beta, and Delta were observed in relation to Omicron BA.1, and all donors exhibited activity against the panel of VOCs tested here (Figure 1E). Compared with unvaccinated individuals, vaccinated individuals infected with Omicron BA.1 displayed significantly higher levels of ADCP, mirroring the binding antibodies (Figures 1E and 1F).

In contrast to binding and ADCP, ADCC in unvaccinated individuals showed significant losses against D614G (3-fold loss) and Beta (4-fold loss). However, like ADCP and binding antibodies, ADCC activity against Delta was retained (Figure 1G). In this group, Omicron BA.1-triggered ADCC was undetectable against D614G and Beta in 25% and 30% of plasma samples, respectively. After previous vaccination, Omicron BA.1 breakthrough infections resulted in overall preservation of ADCC against VOCs, such that only one individual showed undetectable activity against Delta (Figure 1H). Levels of ADCC in previously vaccinated donors were significantly higher than those in unvaccinated individuals, except that ADCC activity against Delta was similar between both groups (Figure 1I).

Lastly, we measured neutralizing antibody responses to Omicron BA.1 and assessed cross-reactivity for VOCs. In addition to testing the variants used above, we also tested C.1.2, a variant that has several neutralization-evasive mutations and circulated at low levels during the third and fourth COVID-19 waves in South

(C) Bars show geometric mean (GM) binding titers for vaccinated (black) and unvaccinated (white) individuals against VOCs.

(D and E) Antibody-dependent cellular phagocytosis (ADCP) of (D) unvaccinated and (E) vaccinated individuals is represented as the percentage of monocytic cells that take up spike-coated beads (D614G, Beta, Delta, and Omicron BA.1) multiplied by their GM fluorescence intensity (MFI).

(F) Bars show GM ADCP scores for vaccinated (black) and unvaccinated (white) individuals against VOCs.

(G and H) Antibody-dependent cellular cytotoxicity (ADCC) in (G) unvaccinated and (H) vaccinated individuals is shown as the relative light units (RLU) signaling through FcγRIIIa-expressing cells.

(I) Bars show GM activity for vaccinated (black) and unvaccinated (white) individuals against VOCs.

All data are representative of two independent experiments. For dot plots, lines indicate GM titer (GMT), which is also represented below the plot with fold decrease and knockout (K/O) of activity for other variants as a percentage relative to Omicron BA.1. Dotted lines indicate the limit of detection of the particular assay. For bar charts, bars indicate the median of function, error bars show standard deviations, and fold decreases relative to vaccinated individuals are indicated below the plot. Statistical significance across variants is shown by the Friedman test with Dunn's correction, and statistical significance between vaccinated and unvaccinated samples is shown by the Mann Whitney test. *p < 0.05, **p < 0.01, ***p < 0.001, ****p < 0.0001, and ns = non-significant.

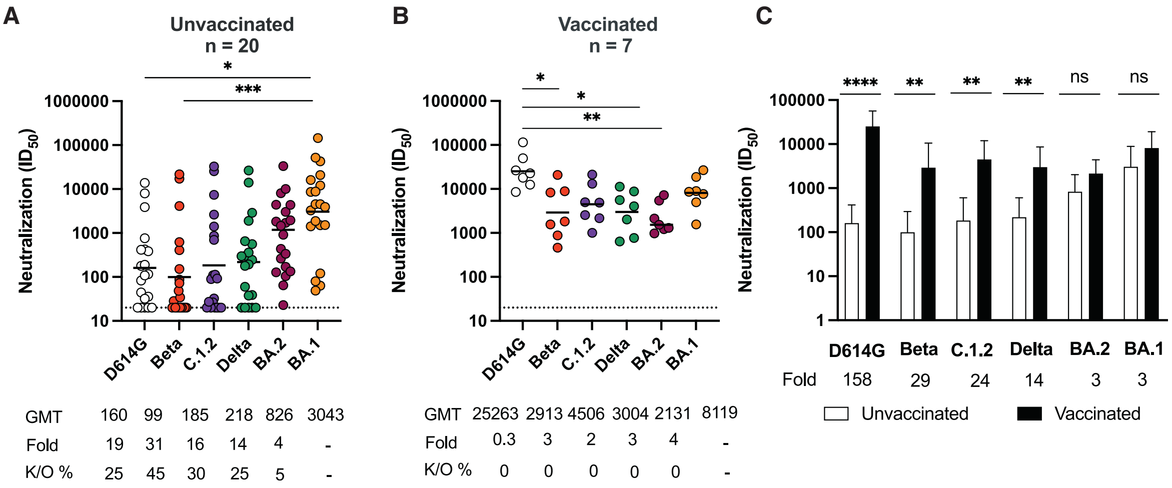

Figure 2. Omicron triggers cross-variant neutralizing antibodies, which are broadened by vaccination

(A and B) The neutralization titer (ID_{50}) of Omicron-infected plasma against D614G, Beta, C.1.2, Delta, Omicron BA.2, and Omicron BA.1 pseudoviruses is shown for (A) unvaccinated individuals (n = 20) or (B) individuals vaccinated with either one dose of Ad26.CoV.2S or two doses of BNT162b2 (n = 7). Lines indicate GMT, which is also represented below the plot with fold decrease and knockout (K/O) of activity for other variants as a percentage relative to Omicron BA.1. Dotted lines indicate the limit of detection of the assay. Statistical significance across variants is shown by the Friedman test with Dunn's correction.

(C) Bars show GM neutralization titers for vaccinated (black) and unvaccinated (white) individuals against VOCs, error bars show standard deviations, and fold decreases relative to vaccinated individuals are indicated below the plot. Statistical significance between vaccinated and unvaccinated samples is shown by the Mann Whitney test.

*$p < 0.05$, **$p < 0.01$, ***$p < 0.001$, ****$p < 0.0001$, and ns = non-significant. All data are representative of two independent experiments.

Africa (Scheepers et al., 2021), and BA.2, a sub-lineage of Omicron. Against Omicron BA.1, unvaccinated individuals showed potent neutralization with a GMT of 3,043. Neutralization of BA.2 was 4-fold lower than that of BA.1 with a GMT of 826, and only 5% of samples showed knockout of neutralization. However, against VOCs, we saw dramatic 19-, 31-, 16-, and 14-fold titer reductions for D614G, Beta, C.1.2, and Delta, respectively, and knockouts ranging from 27% to 45% of plasma tested (Figure 2A), particularly for Beta. This preferential neutralization of Omicron (the infecting strain) over other VOCs is in sharp contrast to samples from the previous three waves of infection in South Africa (for which the infecting virus was D614G, Beta, or Delta), where neutralizing activity against D614G was substantially higher than that against Omicron BA.1 (Figures S1A and S1B). Similarly, samples from BNT162b2 or Ad26.COV2.S showed higher activity against D614G than against Omicron (Figures S1A and S1B), consistent with previous studies (Cele et al., 2021; Garcia-Beltran et al., 2022).

This loss of neutralization was mitigated by prior vaccination, such that all seven breakthrough infections resulted in a significantly increased GMT of 8,119 against Omicron BA.1, a GMT of 2,131 against Omicron BA.2, and high titers against all VOCs (25,263 for D614G; 2,913 for Beta; 4,506 against C.1.2; and 3,004 against Delta) (Figures 2B and 2C). These greatly enhanced titers resulted in far greater increases between previously vaccinated individuals and unvaccinated individuals (158-, 29-, 24-, 14-, 3-, and 3-fold for D614G, Beta, C.1.2, Delta, Omicron BA.2, and Omicron BA.1 respectively) than those seen for Fc effector functions and binding, which ranged from 1- to 3-fold (Figures 1C, 1F, and 1I). Notably, Omicron infection elicited robust and similar neutralization titers against itself regardless of vaccination status.

DISCUSSION

Although the neutralization resistance of Omicron is well defined, here we assess how effectively Omicron-elicited antibodies target D614G and other VOCs. We also measured neutralization activity against BA.2, which now accounts for most infections in South Africa and is increasing globally. We show that in previously unvaccinated individuals, Omicron-triggered antibodies bind and perform Fc effector function with only a slight loss against VOCs. However, with the exception of Omicron BA.2, which showed comparatively modest decreases, VOCs significantly compromised neutralization, indicating limited neutralization cross-reactivity of antibodies elicited by Omicron. In contrast, vaccinated individuals who subsequently became infected with Omicron showed greatly improved cross-reactivity with high titers against Omicron BA.1, BA.2, D614G (one amino acid different from the vaccine spike), Beta, Delta, and C.1.2.

We and others have shown that Fc effector function is largely preserved against VOCs in both convalescent and vaccine-elicited plasma (Kaplonek et al., 2022; Richardson et al., 2022). Also, as with neutralization, we have shown that Fc effector function triggered by Beta is more cross-reactive than antibodies elicited by D614G, indicating that the spike sequence of the eliciting immunogen affects the extent of ADCC cross-reactivity (Moyo-Gwete et al., 2021; Richardson et al., 2022). Here, we show that Omicron infection similarly triggers differential ADCC cross-reactivity: significantly decreased activity against D614G and Beta but not against Delta. This observation extends to vaccinated individuals, in whom ADCC was still significantly poorer against Beta. This differential targeting of ADCC-mediating antibodies indicates that they might preferentially bind sites that differ between Omicron and other VOCs. Alternatively,

different VOCs might trigger antibodies with varied glycosylations and isotypes, both of which modulate Fc effector function (Jennewein and Alter, 2017).

This differential immune imprinting by VOCs was also confirmed for neutralization in this study in both unvaccinated and vaccinated Omicron-infected individuals. The highest titers in unvaccinated individuals were to the infection-matched Omicron. In contrast, in previously vaccinated individuals with breakthrough Omicron infections, high titers were observed against both D614G and Omicron, sequences that match the vaccine and infecting spike proteins to which these donors were exposed. This is consistent with our studies of immune responses triggered by D614G, Beta, or Delta (Keeton et al., 2021; Moyo-Gwete et al., 2021; Wibmer et al., 2021), which resulted in cross-reactivity patterns distinct from those observed here after Omicron infection. Together, these data indicate that the sequence of the infecting spike affects the quality of neutralization, suggesting imprinting of the immune response (Keeton et al., 2021).

We and others have shown that humoral function is significantly boosted in individuals with breakthrough infections after vaccination (Kitchin et al., 2022; Suryawanshi et al., 2022; Walls et al., 2022). This study confirms that this is also true of Omicron breakthrough infections, as shown by 153-fold higher titers to D614G in vaccinated than in unvaccinated individuals, consistent with other studies (Khan et al., 2022; Suryawanshi et al., 2022; Zhou et al., 2022). We also extend our previous study (in which ADCC was boosted by breakthrough infection) to include ADCP, confirming that this applies to other Fc effector functions (Kitchin et al., 2022).

In the absence of vaccination, Omicron-elicited humoral responses, although potent against the matched Omicron spike, show significantly less activity against VOCs. Thus, although highly immunogenic, Omicron does not elicit cross-neutralizing responses. This is consistent with a decreased ability of plasma from unvaccinated individuals to neutralize Delta compared with Omicron after Omicron infection (Khan et al., 2022), which could leave this unvaccinated group at risk of being reinfected with other variants that continued to circulate and evolve in South Africa at the time of this study, including Beta, Delta, and C.1.2. However, we noted only modestly lower neutralizing titers against Omicron BA.2 than against Omicron BA.1 in this cohort, which is in line with a study showing a 3-fold loss in activity against Omicron BA.2 in Omicron BA.1-infected hamsters (Yamasoba et al., 2022). This indicates that despite a number of differences between the sub-lineages, these changes do not seem to greatly alter the capacity of Omicron BA.1 antibodies to neutralize Omicron BA.2.

Our data also have implications for the design of second-generation vaccines based on Omicron, suggesting that these might not trigger cross-reactive *de novo* responses in SARS-CoV-2-naive individuals. This is supported by immunogenicity studies where Delta-infected mice elicited broadly protective antibodies but where Omicron-infected mice failed to mount responses against other VOCs (Suryawanshi et al., 2022). In addition, immunization of mice with an RBD-based Omicron mRNA vaccine elicited only strain-specific neutralization (Lee et al., 2022). In support of this study and others (Hawman et al., 2022), our data also suggest that Omicron is highly immunogenic in that it elicits comparable neutralization titers irrespectively of vaccination status but through a largely strain-specific response. Given the significantly higher titers we see against Omicron in vaccinated individuals, Omicron boosters could be effective in seropositive individuals, a group that exceeds 70% in South Africa (Madhi et al., 2022). However, in a comparison of mRNA-1273-vaccinated rhesus macaques boosted with either mRNA-Omicron or mRNA-1273, Omicron-boosted animals showed lower titers than those with a homologous mRNA-1273 boost (Gagne et al., 2022). Overall, these data suggest that boosting individuals with or without immunity with vaccines specific to Omicron is unlikely to be superior to existing regimens.

Limitations of the study

Our study is limited by the fact that we cannot rule out prior asymptomatic infection, which could alter the quality of humoral responses. Sampling for this study was limited to blood and occurred early in infection when antibodies might not have reached peak cross-reactivity. Furthermore, viral sequences confirming Omicron BA.1 infection were available only for a subset of samples. However, Omicron BA.1 overwhelmingly dominated infections during the wave in which these individuals were tested (Viana et al., 2022). Further, the median age of individuals in this study (58 years) is high and could contribute to the compromised cross-reactivity in unvaccinated individuals. Given the high prevalence of global seropositivity through vaccination or previous infection, the ability to measure the response to Omicron infection in naive samples is limited. As such, our study offers valuable insights into the value of Omicron as an immunogen and the risk of reinfection in unvaccinated individuals.

STAR★METHODS

Detailed methods are provided in the online version of this paper and include the following:

- KEY RESOURCES TABLE
- RESOURCE AVAILABILITY
  - Lead contact
  - Materials availability
  - Data and code availability
- EXPERIMENTAL MODEL AND SUBJECT DETAILS
  - Human subjects
  - Cell lines
- METHOD DETAILS
  - SARS-CoV-2 spike genome sequencing
  - SARS-CoV-2 antigens
  - SARS-CoV-2 spike ELISA
  - Spike plasmid and lentiviral pseudovirus production
  - Pseudovirus neutralization assay
  - ADCP assay
  - ADCC assay
- QUANTIFICATION AND STATISTICAL ANALYSIS

SUPPLEMENTAL INFORMATION

Supplemental information can be found online at https://doi.org/10.1016/j.chom.2022.03.029.

ACKNOWLEDGMENTS

We acknowledge the participants who volunteered for this study and thank W. van Hougenhouck-Tulleken and R. Ramlall for database support and participant recruitment and D. Amoako and J. Giandhari for sequencing. The parental soluble spike was provided by J. McLellan (University of Texas), and parental pseudovirus plasmids were provided by Drs. E. Landais and D. Sok (International AIDS Vaccine Initiative). P.L.M. is supported by the South African Research Chairs Initiative of the Department of Science and Innovation, the National Research Foundation of South Africa, the South African Medical Research Council Strategic Health Innovation Partnerships (SHIP) program, and the Centre for the AIDS Programme of Research in South Africa (CAPRISA). We acknowledge funding from the Bill and Melinda Gates Foundation through the Global Immunology and Immune Sequencing for Epidemic Response (GIISER) program. S.I.R. is a L'Oreal/UNESCO Women in Science South Africa Young Talents awardee.

AUTHOR CONTRIBUTIONS

S.I.R. designed the study, performed experiments, analyzed the data, and wrote the manuscript; N.P.M. and H.S. performed Fc experiments and analyzed data; V.S.M., N.M., and T.M. performed neutralization assays; B.E.L., B.O., and F.A. produced spikes and variant plasmids; F.A. and Z.M. performed ELISA assays supervised by T.M.-G.; M.A.v.d.M. processed samples recruited by Z.d.B., T.R.d.V., A.B., and G.v.d.B.; V.U., T.M.R., and M.T.B. established the Pretoria COVID-19 study, which provided participant samples from the Tshwane District Hospital; A.S., A.M., H.T., M.V., and T.d.O. performed SARS-CoV-2 sequencing; and P.L.M. conceptualized the study and wrote the manuscript.

DECLARATION OF INTERESTS

The authors declare no competing interests.

Received: February 11, 2022
Revised: March 11, 2022
Accepted: March 22, 2022
Published: March 24, 2022

REFERENCES

Ackerman, M.E., Moldt, B., Wyatt, R.T., Dugast, A.-S., McAndrew, E., Tsoukas, S., Jost, S., Berger, C.T., Sciaranghella, G., Liu, Q., et al. (2011). A robust, high-throughput assay to determine the phagocytic activity of clinical antibody samples. J. Immunol. Methods *366*, 8–19. https://doi.org/10.1016/j.jim.2010.12.016.

Bartsch, Y., Tong, X., Kang, J., Avendaño, M.J., Serrano, E.F., García-Salum, T., Pardo-Roa, C., Riquelme, A., Medina, R.A., and Alter, G. (2021). Preserved Omicron spike specific antibody binding and Fc-recognition across COVID-19 vaccine platforms. Preprint at medRxiv. https://doi.org/10.1101/2021.12.24.21268378.

Carreño, J.M., Alshammary, H., Tcheou, J., Singh, G., Raskin, A., Kawabata, H., Sominsky, L., Clark, J., Adelsberg, D.C., Bielak, D., et al. (2021). Activity of convalescent and vaccine serum against SARS-CoV-2 Omicron. Nature *602*, 682–688. https://doi.org/10.1038/d41586-021-03846-z.

Cele, S., Jackson, L., Khoury, D.S., Khan, K., Moyo-Gwete, T., Tegally, H., San, J.E., Cromer, D., Scheepers, C., Amoako, D.G., et al. (2021). Omicron extensively but incompletely escapes Pfizer BNT162b2 neutralization. Nature *602*, 654–656. https://doi.org/10.1038/d41586-021-03824-5.

Collie, S., Champion, J., Moultrie, H., Bekker, L.-G., and Gray, G. (2022). Effectiveness of BNT162b2 vaccine against omicron variant in South Africa. N. Engl. J. Med. *386*, 494–496. https://doi.org/10.1056/nejmc2119270.

Gagne, M., Moliva, J.I., Foulds, K.E., Andrew, S.F., Flynn, B.J., Werner, A.P., Wagner, D.A., Teng, I.-T., Lin, B.C., Moore, C., et al. (2022). mRNA-1273 or mRNA-Omicron boost in vaccinated macaques elicits comparable B cell expansion, neutralizing antibodies and protection against Omicron. Preprint at bioRxiv. https://doi.org/10.1101/2022.02.03.479037.

Gao, Y., Cai, C., Grifoni, A., Müller, T.R., Niessl, J., Olofsson, A., Humbert, M., Hansson, L., Österborg, A., Bergman, P., et al. (2022). Ancestral SARS-CoV-2-specific T cells cross-recognize the Omicron variant. Nat. Med. *28*, 472–476. https://doi.org/10.1038/s41591-022-01700-x.

Garcia-Beltran, W.F., St. Denis, K.J., Hoelzemer, A., Lam, E.C., Nitido, A.D., Sheehan, M.L., Berrios, C., Ofoman, O., Chang, C.C., Hauser, B.M., et al. (2022). mRNA-based COVID-19 vaccine boosters induce neutralizing immunity against SARS-CoV-2 Omicron variant. Cell *185*, 457–466.e4. https://doi.org/10.1016/j.cell.2021.12.033.e4.

Hadfield, J., Megill, C., Bell, S.M., Huddleston, J., Potter, B., Callender, C., Sagulenko, P., Bedford, T., and Neher, R.A. (2018). Nextstrain: Real-time tracking of pathogen evolution. Bioinformatics *34*, 4121–4123. https://doi.org/10.1093/bioinformatics/bty407.

Hawman, D.W., Meade-White, K., Clancy, C., Archer, J., Hinkley, T., Leventhal, S.S., Rao, D., Stamper, A., Lewis, M., Rosenke, R., et al. (2022). Replicating RNA platform enables rapid response to the SARS-CoV-2 Omicron variant and elicits enhanced protection in naïve hamsters compared to ancestral vaccine. Preprint at bioRxiv. https://doi.org/10.1101/2022.01.31.478520.

Jennewein, M.F., and Alter, G. (2017). The immunoregulatory roles of antibody glycosylation. Trends Immunol. *38*, 358–372. https://doi.org/10.1016/j.it.2017.02.004.

Kaplonek, P., Fischinger, S., Cizmeci, D., Bartsch, Y.C., Kang, J., Burke, J.S., Shin, S.A., Dayal, D., Martin, P., Mann, C., et al. (2022). mRNA-1273 vaccine-induced antibodies maintain Fc effector functions across SARS-CoV-2 variants of concern. Immunity *55*, 355–365.e4. https://doi.org/10.1016/j.immuni.2022.01.001.

Keeton, R., Richardson, S.I., Moyo-Gwete, T., Hermanus, T., Tincho, M.B., Benede, N., Manamela, N.P., Baguma, R., Makhado, Z., Ngomti, A., et al. (2021). Prior infection with SARS-CoV-2 boosts and broadens Ad26.COV2.S immunogenicity in a variant-dependent manner. Cell Host Microbe *29*, 1611–1619.e5. https://doi.org/10.1016/j.chom.2021.10.003.e5.

Keeton, R., Tincho, M.B., Ngomti, A., Baguma, R., Benede, N., Suzuki, A., Khan, K., Cele, S., Bernstein, M., Karim, F., et al. (2022). T cell responses to SARS-CoV-2 spike cross-recognize Omicron. Nature *603*, 488–492. https://doi.org/10.1038/s41586-022-04460-3.

Khan, K., Karim, F., Cele, S., San, J.E., Lustig, G., Tegally, H., Rosenberg, Y., Bernstein, M., Ganga, Y., Jule, Z., et al. (2022). Omicron infection of vaccinated individuals enhances neutralizing immunity against the Delta variant. Preprint at medRxiv. https://doi.org/10.1101/2021.12.27.21268439.

Kitchin, D., Richardson, S.I., van der Mescht, M.A., Motlou, T., Mzindle, N., Moyo-Gwete, T., Makhado, Z., Ayres, F., Manamela, N.P., Spencer, H., et al. (2022). Ad26.COV2.S breakthrough infections induce high titers of neutralizing antibodies against Omicron and other SARS-CoV-2 variants of concern. Cell Rep. Med. *3*, 100535. https://doi.org/10.1016/j.xcrm.2022.100535.

Lee, I.-J., Sun, C.-P., Wu, P.-Y., Lan, Y.-H., Wang, I.-H., Liu, W.-C., Tseng, S.-C., Tsung, S.-I., Chou, Y.-C., Kumari, M., et al. (2022). Omicron-specific mRNA vaccine induced potent neutralizing antibody against Omicron but not other SARS-CoV-2 variants. Preprint at bioRxiv. https://doi.org/10.1101/2022.01.31.478406.

Madhi, S.A., Kwatra, G., Myers, J.E., Jassat, W., Dhar, N., Mukendi, C.K., Nana, A.J., Blumberg, L., Welch, R., Ngorima-Mabhena, N., et al. (2022). South African population immunity and severe covid-19 with omicron variant. Preprint at medRxiv. https://doi.org/10.1101/2021.12.20.21268096.

Moyo-Gwete, T., Madzivhandila, M., Makhado, Z., Ayres, F., Mhlanga, D., Oosthuysen, B., Lambson, B.E., Kgagudi, P., Tegally, H., Iranzadeh, A., et al. (2021). Cross-reactive neutralizing antibody responses elicited by SARS-CoV-2 501Y.V2 (B.1.351). N. Engl. J. Med. *384*, 2161–2163. https://doi.org/10.1056/nejmc2104192.

Richardson, S.I., Manamela, N.P., Motsoeneng, B.M., Kaldine, H., Ayres, F., Makhado, Z., Mennen, M., Skelem, S., Williams, N., Sullivan, N.J., et al. (2022). SARS-CoV-2 Beta and Delta variants trigger Fc effector function with increased cross-reactivity. Cell Rep. Med. *3*, 100510. https://doi.org/10.1016/j.xcrm.2022.100510.

Scheepers, C., Everatt, J., Amoako, D.G., Tegally, H., Wibmer, C.K., Mnguni, A., Ismail, A., Mahlangu, B., Lambson, B.E., Richardson, S.I., et al. (2021). Emergence and phenotypic characterization of C.1.2, a globally detected lineage that rapidly accumulated mutations of concern. Preprint at medRxiv. https://doi.org/10.1101/2021.08.20.21262342.

Schmidt, F., Muecksch, F., Weisblum, Y., Da Silva, J., Bednarski, E., Cho, A., Wang, Z., Gaebler, C., Caskey, M., Nussenzweig, M.C., et al. (2021). Plasma neutralization of the SARS-CoV-2 omicron variant. Preprint at N. Engl. J. Med. https://doi.org/10.1101/2021.12.12.21267646.

Sievers, B.L., Chakraborty, S., Xue, Y., Gelbart, T., Gonzalez, J.C., Cassidy, A.G., Golan, Y., Prahl, M., Gaw, S.L., Arunachalam, P.S., et al. (2022). Antibodies elicited by SARS-CoV-2 infection or mRNA vaccines have reduced neutralizing activity against Beta and Omicron pseudoviruses. Sci. Transl. Med. *14*, eabn7842. https://doi.org/10.1126/scitranslmed.abn7842.

Suryawanshi, R.K., Chen, I.P., Ma, T., Syed, A.M., Simoneau, C.R., Ciling, A., Khalid, M.M., Sreekumar, B., Chen, P.-Y., George, A.F., et al. (2022). Limited cross-variant immunity after infection with the SARS-CoV-2 Omicron variant without vaccination. Preprint at medRxiv. https://doi.org/10.1101/2022.01.13.22269243.

Tarke, A., Coelho, C.H., Zhang, Z., Dan, J.M., Yu, E.D., Methot, N., Bloom, N.I., Goodwin, B., Phillips, E., Mallal, S., et al. (2022). SARS-CoV-2 vaccination induces immunological T cell memory able to cross-recognize variants from Alpha to Omicron. Cell *185*, 847–859.e11. https://doi.org/10.1016/j.cell.2022.01.015.

Tegally, H., Wilkinson, E., Giovanetti, M., Iranzadeh, A., Fonseca, V., Giandhari, J., Doolabh, D., Pillay, S., San, E.J., Msomi, N., et al. (2021). Detection of a SARS-CoV-2 variant of concern in South Africa. Nature *592*, 438–443. https://doi.org/10.1038/s41586-021-03402-9.

Viana, R., Moyo, S., Amoako, D.G., Tegally, H., Scheepers, C., Althaus, C.L., Anyaneji, U.J., Bester, P.A., Boni, M.F., Chand, M., et al. (2022). Rapid epidemic expansion of the SARS-CoV-2 Omicron variant in southern Africa. Nature *603*, 679–686. https://doi.org/10.1038/d41586-021-03832-5.

Walls, A.C., Sprouse, K.R., Bowen, J.E., Joshi, A., Franko, N., Navarro, M.J., Stewart, C., Cameroni, E., McCallum, M., Goecker, E.A., et al. (2022). SARS-CoV-2 breakthrough infections elicit potent, broad, and durable neutralizing antibody responses. Cell *85*, 872–880.e3. https://doi.org/10.1016/j.cell.2022.01.011.

Wibmer, C.K., Ayres, F., Hermanus, T., Madzivhandila, M., Kgagudi, P., Oosthuysen, B., Lambson, B.E., de Oliveira, T., Vermeulen, M., van der Berg, K., et al. (2021). SARS-CoV-2 501Y.V2 escapes neutralization by South African COVID-19 donor plasma. Nat. Med. *27*, 622–625. https://doi.org/10.1038/s41591-021-01285-x.

Yamasoba, D., Kimura, I., Nasser, H., Morioka, Y., Nao, N., Ito, J., Uriu, K., Tsuda, M., Zahradnik, J., Shirakawa, K., et al. (2022). Virological characteristics of SARS-CoV-2 BA.2 variant. Preprint at bioRxiv. https://doi.org/10.1101/2022.02.14.480335.

Yu, J., Tostanoski, L.H., Peter, L., Mercado, N.B., McMahan, K., Mahrokhian, S.H., Nkolola, J.P., Liu, J., Li, Z., Chandrashekar, A., et al. (2020). DNA vaccine protection against SARS-CoV-2 in rhesus macaques. Science *369*, 806–811. https://doi.org/10.1126/science.abc6284.

Yu, J., Collier, A.-R.Y., Rowe, M., Mardas, F., Ventura, J.D., Wan, H., Miller, J., Powers, O., Chung, B., Siamatu, M., et al. (2022). Comparable neutralization of the SARS-CoV-2 omicron BA.1 and BA.2 variants. Preprint at medRxiv. https://doi.org/10.1101/2022.02.06.22270533.

Zhou, R., To, K.K.-W., Peng, Q., Chan, J.M.-C., Huang, H., Yang, D., Lam, B.H.-S., Chuang, V.W.-M., Cai, J.-P., Liu, N., et al. (2022). Vaccine-breakthrough infection by the SARS-CoV-2 omicron variant elicits broadly cross-reactive immune responses. Clin. Transl. Med. *12*, e720. https://doi.org/10.1002/ctm2.720.

Zohar, T., Loos, C., Fischinger, S., Atyeo, C., Wang, C., Slein, M.D., Burke, J., Yu, J., Feldman, J., Hauser, B.M., et al. (2020). Compromised humoral functional evolution tracks with SARS-CoV-2 mortality. Cell *183*, 1508–1519.e12. https://doi.org/10.1016/j.cell.2020.10.052.e12.

STAR★METHODS

KEY RESOURCES TABLE

REAGENT or RESOURCE	SOURCE	IDENTIFIER
Antibodies		
CR3022	GenScript (https://www.genscript.com)	N/A
P2B-2F6	Dr. Nicole Doria-Rose, VRC, USA	N/A
084-7D	this manuscript	N/A
anti-IgG APC (clone QA19A42)	BioLegend	Cat # 366905; RRID: AB_2888847
Palivizumab	Medimmune	Synagis; RRID: AB_2459638
Bacterial and virus strains		
SARS-CoV-2 pseudoviruses for ancestral, Beta, Delta, Omicron BA.1, C.1.2, Omicron BA.2	Wibmer et al. (2021), Keeton et al. (2021), Scheepers et al. (2021), this manuscript	N/A
Biological samples		
Convalescent hospitalized blood samples	Groote Schuur Hospital	https://www.gsh.co.za
Convalescent hospitalized blood samples	Steve Biko Academic Hospital	https://www.sbah.org.za
Ad26.COV2.S vaccinee blood samples	National institute for Communicable Diseases	https://www.nicd.ac.za
BNT162b2 vaccinee blood samples	National institute for Communicable Diseases	https://www.nicd.ac.za
Chemicals, peptides, and recombinant proteins		
SARS-CoV-2 original and Beta variant spike proteins	original: Dr. Jason McKellan; Beta: Moyo-Gwete et al. (2021)	N/A
Critical commercial assays		
PEI-MAX 40,000	Polysciences	Cat # 24765–1
QUANTI-Luc luciferase	Invivogen	Cat # rep-qlc2
EZ link Sulfo-NHS-LC-Biotin kit	ThermoFisher	Cat # 21435
FluoSpheres NeutrAvidin-Labeled Microspheres, 1.0 μm	ThermoFisher	Cat # F8776
Luciferase	Promega	Cat # PRE263B-C
Experimental models: Cell lines		
Human embryonic kidney (HEK) 293F	Dr. Nicole Doria-Rose, VRC, USA	N/A
HEK 293T/ACE2.MF	Dr. Michael Farzan, Scripps, USA	N/A
Jurkat-Lucia NFAT-CD16 cells	Invivogen	Cat # jktl-nfat-cd16
HEK 293T cells	Dr. George Shaw, UPenn, USA	N/A
THP-1 cells	NIH HIV Reagent program	Cat # ARP-9942
Recombinant DNA		
Spike Hexapro plasmid	original: Dr. Jason McKellan; Beta: Moyo-Gwete et al. (2021)	N/A
SARS-CoV-2 ancestral variant spike (D614G) plasmid	Wibmer et al. (2021)	N/A
Beta spike (L18F, D80A, D215G, K417N, E484K, N501Y, D614G, A701V, 242–244 del) plasmid	Wibmer et al. (2021)	N/A
Delta spike (T19R, R158G L452R, T478K, D614G, P681R, D950N, 156–157 del) plasmid	Keeton et al. (2021)	N/A
Omicron BA.1 plasmid (A67V, Δ69-70, T95I, G142D, Δ143-145, Δ211, L212I, 214EPE, G339D, S371L, S373P, S375F, K417N, N440K, G446S, S477N, T478K, E484A, Q493R, G496S, Q498R, N501Y, Y505H, T547K, D614G, H655Y, N679K, P681H, N764K, D796Y, N856K, Q954H, N969K, L981F)	this manuscript	N/A

(*Continued on next page*)

Continued		
REAGENT or RESOURCE	SOURCE	IDENTIFIER
C.1.2 plasmid (P9L, P25L, C136F, Δ144, R190S, D215G, Δ242-243, Y449H, E484K, N501Y, L585F, D614G, H655Y, N679K, T716I, T859N)	Scheepers et al. (2021)	N/A
Omicron BA.2 plasmid (T19I, L24S, 25–27del, G142D, V213G, G339D, S371F, S373P, S375F, T376A, D405N, R408S,K417N,N440K, S477N, T478K, E484A, Q493R, Q498R, N501Y, Y505H, D614G, H655Y, N679K, P681H, N764K, D796Y, Q954H, N969K)	this manuscript	N/A
Firefly luciferase encoding lentivirus backbone plasmid	Dr. Michael Farzan, Scripps	N/A
Software and algorithms		
Genome Detective 1.132	Genome Detective	https://www.genomedetective.com
Geneious software	Biomatters	https://www.geneious.com
NextStrain	Hadfield et al. (2018)	https://github.com/nextstrain/ncov
FACSDiva 9	BD Biosciences	https://www.bdbiosciences.com
FlowJo 10	FlowJo	https://www.flowjo.com
GraphPad Prism 9	GraphPad	https://www.graphpad.com
BioRender	BioRender	https://www.biorender.com

RESOURCE AVAILABILITY

Lead contact

Further information and reasonable requests for resources and reagents should be directed to and will be fulfilled by the lead contact, Penny Moore (pennym@nicd.ac.za).

Materials availability

Materials will be made by request to Penny Moore (pennym@nicd.ac.za).

Data and code availability

- All data reported in this paper will be shared by the lead contact upon request.
- This paper does not report any original code.
- Any additional information required for reanalyzing the data reported in this paper is available from the lead contact upon request.

EXPERIMENTAL MODEL AND SUBJECT DETAILS

Human subjects

Samples infected in the fourth COVID-19 wave of infection in South Africa were collected from participants enrolled to the Pretoria COVID-19 study cohort. Participants were admitted to Tshwane District Hospital (Pretoria, South Africa) with mild to severe (WHO severity scale 3–6) PCR confirmed SARS-CoV-2 infection between 25 November 2021–20 December 2021 (Table S1). Ethics approval was received from the University of Pretoria, Human Research Ethics Committee (Medical) (247/2020). Second wave plasma samples were obtained from hospitalized COVID-19 patients with moderate disease (WHO scale 4–5) admitted to Groote Schuur Hospital cohort, Cape Town from December 2020 to January 2021. This study received ethics approval from the Human Research Ethics Committee of the Faculty of Health Sciences, University of Cape Town (R021/2020). Wave 3 samples were collected from hospitalized COVID-19 patients with moderate disease (WHO scale 4–5) from Steve Biko Academic Hospital and Groote Schuur Hospital in July 2021. All patients had PCR-confirmed SARS-CoV-2 infection before blood collection, which was done a median of 4 days post positive PCR test. Healthy donors who were confirmed to be SARS-CoV-2 infection naive and were vaccinated with either one dose of Janssen/Johnson and Johnson (Ad26.COV2.S) vaccine during the Sisonke Trial or two doses of BNT162b2 were obtained 2 months after full vaccination. Ethics approval for the use of these samples were obtained from the Human Research Ethics Committee of the Faculty of Health Sciences, University of the Witwatersrand (M210465). Written informed consent was obtained from all participants.

Cell lines

Human embryo kidney (HEK) 293T cells were cultured at 37°C and 5% CO_2 in DMEM containing 10% heat-inactivated fetal bovine serum (Gibco BRL Life Technologies) and supplemented with 50 μg/mL gentamicin (Sigma). Cells were disrupted at confluence with

CellPress
OPEN ACCESS

0.25% trypsin in 1 mM EDTA (Sigma) every 48–72 h. HEK 293T/ACE2.MF cells were maintained in the same way as HEK 293T cells but were supplemented with 3 μg/mL puromycin for selection of stably transduced cells. HEK 293F suspension cells were cultured in 293 Freestyle media (Gibco BRL Life Technologies) and cultured in a shaking incubator at 37°C, 5% CO_2, and 70% humidity at 125 rpm maintained between 0.2 and 0.5 million cells/mL. Jurkat-Lucia NFAT-CD16 cells were maintained in IMDM media with 10% heat-inactivated fetal bovine serum (Gibco, Gaithersburg, MD), 1% penicillin streptomycin (Gibco, Gaithersburg, MD) and 10 μg/mL of blasticidin and 100 μg/mL of zeocin was added to the growth medium every other passage. THP-1 cells were used for the ADCP assay and obtained from the AIDS Reagent Program, Division of AIDS, NIAID, NIH contributed by Dr. Li Wu and Vineet N. KewalRamani. Cells were cultured at 37°C and 5% CO_2 in RPMI containing 10% heat-inactivated fetal bovine serum (Gibco, Gaithersburg, MD) with 1% Penicillin Streptomycin (Gibco, Gaithersburg, MD) and 2-mercaptoethanol to a final concentration of 0.05 mM and not allowed to exceed 4×10^5 cells/mL to prevent differentiation.

METHOD DETAILS

SARS-CoV-2 spike genome sequencing

Sequencing of the spike was performed as previously described (Tegally et al., 2021) using swabs obtained from Tshwane District Hospital patients, of which six were available and confirmed to be Omicron BA.1 (Table S1). RNA sequencing was performed as previously published. Briefly, extracted RNA was used to synthesize cDNA using the Superscript IV First Strand synthesis system (Life Technologies, Carlsbad, CA) and random hexamer primers. SARS-CoV-2 whole genome amplification was performed by multiplex PCR using primers designed on Primal Scheme (http://primal.zibraproject.org/) to generate 400 bp amplicons with a 70 bp overlap covering the SARS-CoV-2 genome. Phylogenetic clade classification of the genomes in this study consisted of analyzing them against a global reference dataset using a custom pipeline based on a local version of NextStrain (https://github.com/nextstrain/ncov) (Hadfield et al., 2018).

SARS-CoV-2 antigens

For ELISA and ADCP assays, SARS-CoV-2 original and Beta variant full spike (L18F, D80A, D215G, K417N, E484K, N501Y, D614G, A701V, 242–244 del), Delta (T19R, 156–157del, R158G, L452R, T478K, D614G, P681R and D950N) and Omicron BA.1 (A67V, Δ69-70, T95I, G142D, Δ143-145, Δ211, L212I, 214EPE, G339D, S371L, S373P, S375F, K417N, N440K, G446S, S477N, T478K, E484A, Q493R, G496S, Q498R, N501Y, Y505H, T547K, D614G, H655Y, N679K, P681H, N764K, D796Y, N856K, Q954H, N969K, L981F) proteins were expressed in HEK 293F suspension cells by transfecting the cells with the respective expression plasmid. After incubating for 6 days at 37°C, 70% humidity, and 10% CO_2, proteins were first purified using a nickel resin followed by size-exclusion chromatography. Relevant fractions were collected and frozen at −80°C until use.

SARS-CoV-2 spike ELISA

Two μg/mL of spike protein (D614G, Beta, Delta, or Omicron) was used to coat 96-well, high-binding plates and incubated overnight at 4°C. The plates were incubated in a blocking buffer consisting of 5% skimmed milk powder, 0.05% Tween 20, 1× PBS. Plasma samples were diluted to 1:100 starting dilution in a blocking buffer and added to the plates. IgG secondary antibody was diluted to 1:3000 in blocking buffer and added to the plates followed by TMB substrate (Thermofisher Scientific). Upon stopping the reaction with 1 M H_2SO_4, absorbance was measured at a 450 nm wavelength. In all instances, mAbs CR3022 and BD23 were used as positive controls and Palivizumab was used as a negative control.

Spike plasmid and lentiviral pseudovirus production

The SARS-CoV-2 Wuhan-1 spike, cloned into pCDNA3.1 was mutated using the QuikChange Lightning Site-Directed Mutagenesis kit (Agilent Technologies) and NEBuilder HiFi DNA Assembly Master Mix (NEB) to include D614G (original) or lineage defining mutations for Beta (L18F, D80A, D215G, 242–244del, K417N, E484K, N501Y, D614G and A701V), Delta (T19R, 156–157del, R158G, L452R, T478K, D614G, P681R and D950N), C.1.2 (P9L, P25L, C136F, Δ144, R190S, D215G, Δ242-243, Y449H, E484K, N501Y, L585F, D614G, H655Y, N679K, T716I, T859N), Omicron BA.1 (A67V, Δ69-70, T95I, G142D, Δ143-145, Δ211, L212I, 214EPE, G339D, S371L, S373P, S375F, K417N, N440K, G446S, S477N, T478K, E484A, Q493R, G496S, Q498R, N501Y, Y505H, T547K, D614G, H655Y, N679K, P681H, N764K, D796Y, N856K, Q954H, N969K, L981F) or Omicron BA.2 (T19I, L24S, 25–27del, G142D, V213G, G339D, S371F, S373P, S375F, T376A, D405N, R408S,K417N,N440K, S477N, T478K, E484A, Q493R, Q498R, N501Y, Y505H, D614G, H655Y, N679K, P681H, N764K, D796Y, Q954H, N969K).

Pseudotyped lentiviruses were prepared by co-transfecting HEK 293T cell line with the SARS-CoV-2 ancestral variant spike (D614G), Beta, Delta, C.1.2, Omicron BA.1, or Omicron BA.2 spike plasmids in conjunction with a firefly luciferase encoding lentivirus backbone (HIV-1 pNL4.luc) plasmid as previously described (Wibmer et al., 2021). Culture supernatants were clarified of cells by a 0.45-μM filter and stored at −70°C. Other pcDNA plasmids were used for the ADCC assay.

Pseudovirus neutralization assay

For the neutralization assay, plasma samples were heat-inactivated and clarified by centrifugation. Heat-inactivated plasma samples from vaccine recipients were incubated with the SARS-CoV-2 pseudotyped virus for 1 h at 37°C and 5% CO_2. Subsequently, 1×10^4 HEK 293T cells engineered to over-express ACE-2 (293T/ACE2.MF) (kindly provided by M. Farzan, Scripps Research) were added

and incubated at 37°C and 5% CO_2 for 72 h, upon which the luminescence of the luciferase gene was measured. Titers were calculated as the reciprocal plasma dilution (ID_{50}) causing 50% reduction of relative light units. CB6 and CA1 was used as positive controls for D614G, Beta and Delta. 084-7D, a mAb targeting K417N was used as a positive control for Omicron and Beta.

ADCP assay

Avitagged SARS-CoV-2 spikes were biotinylated using the BirA biotin-protein ligase standard reaction kit (Avidity) and coated onto fluorescent neutravidin beads as previously described (Ackerman et al., 2011). In brief, beads were incubated for two hours with monoclonal antibodies at a starting concentration of 20 μg/mL or plasma at a single 1 in 100 dilution. Opsonized beads were incubated with the monocytic THP-1 cell line overnight, fixed and interrogated on the FACSAria II. Phagocytosis score was calculated as the percentage of THP-1 cells that engulfed fluorescent beads multiplied by the GM fluorescence intensity of the population less the no antibody control. For this and all subsequent Fc effector assays, pooled plasma from 5 PCR-confirmed SARS-CoV-2 infected individuals and CR3022 were used as positive controls and plasma from 5 pre-pandemic healthy controls and Palivizumab were used as negative controls. 084-7D was used as a positive control for Omicron BA.1 and Beta. In addition samples both waves were run head to head in the same experiment. ADCP scores for different spikes were normalized to each other and between runs using CR3022.

ADCC assay

The ability of plasma antibodies to cross-link and signal through FcγRIIIa (CD16) and spike expressing cells or SARS-CoV-2 protein was measured as a proxy for ADCC. For spike assays, HEK 293T cells were transfected with 5 μg of SARS-CoV-2 spike plasmids using PEI-MAX 40,000 (Polysciences) and incubated for 2 days at 37°C. Expression of spike was confirmed by differential binding of CR3022 and P2B-2F6 and their detection by anti-IgG APC staining measured by flow cytometry. Subsequently, 1×10^5 spike transfected cells per well were incubated with heat inactivated plasma (1:100 final dilution) or monoclonal antibodies (final concentration of 100 μg/mL) in RPMI 1640 media supplemented with 10% FBS 1% Pen/Strep (Gibco, Gaithersburg, MD) for 1 h at 37°C. Jurkat-Lucia NFAT-CD16 cells (Invivogen) (2×10^5 cells/well and 1×10^5 cells/well for spike and other protein respectively) were added and incubated for 24 h at 37°C and 5% CO_2. Twenty μL of supernatant was then transferred to a white 96-well plate with 50 μL of reconstituted QUANTI-Luc secreted luciferase and read immediately on a Victor 3 luminometer with 1 s integration time. Relative light units (RLU) of a no antibody control was subtracted as background. Palivizumab was used as a negative control, while CR3022 was used as a positive control, and P2B-2F6 to differentiate the Beta from the D614G variant. 084-7D was used as a positive control for Omicron BA.1 and Beta. To induce the transgene 1× cell stimulation cocktail (Thermofisher Scientific, Oslo, Norway) and 2 μg/mL ionomycin in R10 was added as a positive control to confirm sufficient expression of the Fc receptor. RLUs for spikes were normalized to each other and between runs using CR3022. All samples were run head to head in the same experiment as were all variants tested.

QUANTIFICATION AND STATISTICAL ANALYSIS

Analyses were performed in Prism (v9; GraphPad Software, San Diego, CA, USA). Non-parametric tests were used for all comparisons. The Mann-Whitney and Wilcoxon tests were used for unmatched and paired samples, respectively. The Friedman test with Dunn's correction for multiple comparisons was used for matched comparisons across variants. All correlations reported are non-parametric Spearman's correlations. p values less than 0.05 were considered to be statistically significant.

Cell Host & Microbe, Volume *30*

Supplemental information

SARS-CoV-2 Omicron triggers cross-reactive neutralization and Fc effector functions in previously vaccinated, but not unvaccinated, individuals

Simone I. Richardson, Vimbai Sharon Madzorera, Holly Spencer, Nelia P. Manamela, Mieke A. van der Mescht, Bronwen E. Lambson, Brent Oosthuysen, Frances Ayres, Zanele Makhado, Thandeka Moyo-Gwete, Nonkululeko Mzindle, Thopisang Motlou, Amy Strydom, Adriano Mendes, Houriiyah Tegally, Zelda de Beer, Talita Roma de Villiers, Annie Bodenstein, Gretha van den Berg, Marietjie Venter, Tulio de Oliviera, Veronica Ueckermann, Theresa M. Rossouw, Michael T. Boswell, and Penny L. Moore

Table S1: Demographic and clinical description of the cohort, Related to STAR Methods

Study number	Age	Gender	Positive PCR test	Days sampled post infection	Vaccination status	Days since vaccination	Sequence	Severity	WHO severity scale
COV132	36	Male	2021/11/28	9	Unvaccinated	-	Not sequenced	Severe	6
COV140	29	Female	2021/12/09	1	Unvaccinated	-	Not sequenced	Mild	2
COV145	17	Female	2021/12/03	3	Unvaccinated	-	Omicron	Mild	2
COV149	63	Female	2021/12/05	3	Unvaccinated	-	Not sequenced	Mild	2
COV157	66	Male	2021/12/04	4	Unvaccinated	-	Omicron	Moderate	5
COV158	69	Female	2021/12/14	6	Unvaccinated	-	Not sequenced	Moderate	5
COV159	24	Male	2021/11/25	6	Unvaccinated	-	Not sequenced	Mild	2
COV162	58	Male	2021/12/09	5	Unvaccinated	-	Not sequenced	Moderate	5
COV165	62	Female	2021/12/10	4	Unvaccinated	-	Not sequenced	Moderate	5
COV166	59	Male	2021/12/10	4	Unvaccinated	-	Not sequenced	Severe	6
COV167	62	Female	2021/11/29	8	Unvaccinated	-	Not sequenced	Severe	5
COV176	75	Male	2021/12/20	2	Unvaccinated	-	Not sequenced	Moderate	6
COV178	61	Female	2021/12/19	2	Unvaccinated	-	Not sequenced	Moderate	5
COV183	27	Male	2021/12/12	2	Unvaccinated	-	Not sequenced	Mild	2
COV184	59	Male	2021/12/14	1	Unvaccinated	-	Not sequenced	Moderate	5
COV185	47	Female	2021/12/13	1	Unvaccinated	-	Not sequenced	Moderate	5
COV186	58	Male	2021/12/03	10	Unvaccinated	-	Omicron	Moderate	5
COV187	35	Female	2021/12/11	3	Unvaccinated	-	Not sequenced	Mild	2
COV189	49	Male	2021/12/09	5	Unvaccinated	-	Not sequenced	Moderate	5
COV190	34	Female	2021/12/10	4	Unvaccinated	-	Omicron	Mild	2
COV151	48	Male	2021/12/07	1	One dose Ad26.CoV2.S	56	Omicron	Severe	5
COV182	39	Male	2021/12/10	3	One dose Ad26.CoV2.S	Unknown	Not sequenced	Mild	2
COV131	59	Female	2021/12/10	4	Two doses BNT162b2	109	Not sequenced	Severe	6
COV135	85	Male	2021/11/26	5	Two doses BNT162b2	72	Omicron	Mild	2
COV138	68	Male	2021/11/25	5	Two doses BNT162b2	Unknown	Not sequenced	Moderate	3
COV161	64	Female	2021/12/05	5	Two doses BNT162b2	112	Not sequenced	Mild	2
COV181	71	Male	2021/12/16	4	Two doses BNT162b2	163	Not sequenced	Severe	6

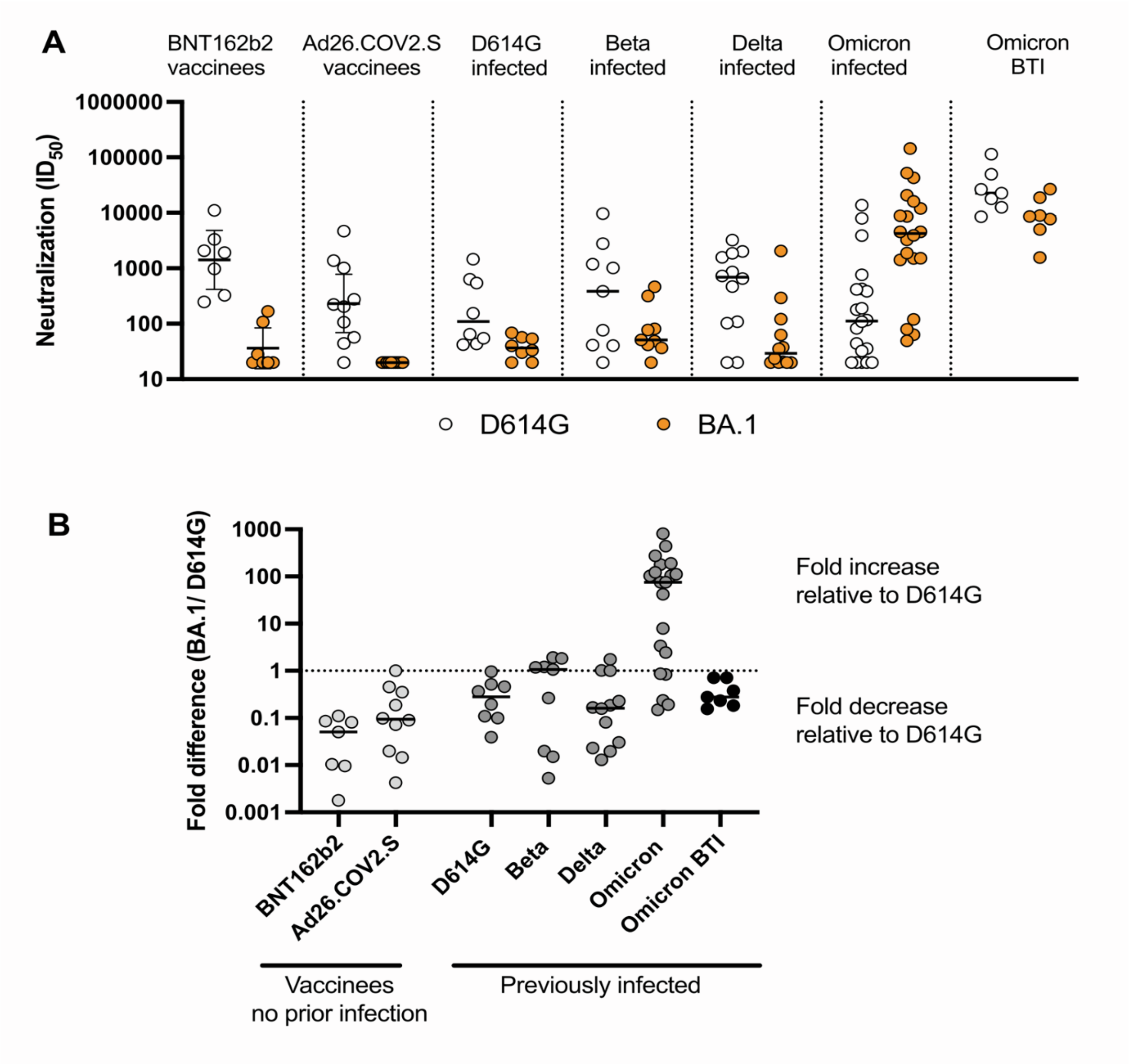

Figure S1: Omicron BA.1 neutralization titers are substantially boosted in response to Omicron infection compared to infection with other VOCs or vaccination, Related to Figure 2

(A) Neutralization ID_{50} titers from vaccinees two months post two-doses of BNT162b2 or one-dose of Ad26.COV2.S, individuals acutely infected with D614G, Beta, Delta or Omicron or individuals who were vaccinated with either BNT162b2 or Ad26.COV2.S and then infected with Omicron (BTI = breakthrough infection) against D614G (white) and Omicron BA.1 (orange) SARS-CoV-2 variants. Lines indicate geometric mean titer (GMT). (B) Fold difference of neutralization titer against Omicron BA.1 relative to D614G for each individual is shown with median fold difference indicated by the bold line. The dotted line indicates no fold difference.
